# Genomic Signature of the Standardized Uptake Value in ^18^F-Fluorodeoxyglucose Positron Emission Tomography in Breast Cancer

**DOI:** 10.3390/cancers12020497

**Published:** 2020-02-20

**Authors:** Seon-Kyu Kim, Sung Gwe Ahn, Jeong-Yeon Mun, Mi-So Jeong, Soong June Bae, Ju-Seog Lee, Joon Jeong, Sun-Hee Leem, In-Sun Chu

**Affiliations:** 1 Personalized Genomic Medicine Research Center, Korea Research Institute of Bioscience and Biotechnology (KRIBB), Daejeon 34141, Korea; seonkyu@kribb.re.kr; 2 Department of Surgery, Gangnam Severance Hospital, Yonsei University College of Medicine, Seoul 06273, Korea; asg2004@yuhs.ac (S.G.A.); mission815815@yuhs.ac (S.J.B.); 3 Department of Biological Science, Dong-A University, Busan 49315, Korea; moonmihye90@hanmail.net (J.-Y.M.); dkwl523@hanmail.net (M.-S.J.); 4 Department of Systems Biology, The University of Texas MD Anderson Cancer Center, Houston, TX 77030, USA; jlee@mdanderson.org; 5 Genome Editing Research Center, KRIBB, Daejeon 34141, Korea; 6 Department of Bioinformatics, KRIBB School of Bioscience, Korea University of Science and Technology, Daejeon 34113, Korea

**Keywords:** FDG-PET, breast cancer, glucose metabolism, Warburg effect, immune checkpoint inhibitor

## Abstract

The standardized uptake value (SUV), an indicator of the degree of glucose uptake in ^18^F-fluorodeoxyglucose positron emission tomography (FDG-PET), has been used for predicting the clinical behavior of malignant tumors. However, its characteristics have been insufficiently explored at the genomics level. Here, we aim to identify genomic signatures reflecting prognostic SUV characteristics in breast cancer (BRC). Through integrative genomic profiling of 3710 BRC patients, including 254 patients who underwent preoperative FDG-PET, we identified an SUV signature, which showed independent clinical utility for predicting BRC prognosis (hazard ratio [HR] 1.27, 95% confidence interval [CI] = 1.12 to 1.45, *p* = 2.23 × 10^−4^). The risk subgroups classified by the signature exhibited mutually exclusive mutation patterns of *TP53* and *PIK3CA* and showed significantly different responsiveness to immunotherapy. Experimental assays revealed that a signaling axis defined by *TP53*–*FOXM1* and its downstream effectors in glycolysis–gluconeogenesis, including *LDHA*, might be important mediators in the FDG-PET process. Our molecular characterizations support an understanding of glucose metabolism and poor prognosis in BRC with a high SUV, utilizable in clinical practice to assist other diagnostic tools.

## 1. Introduction

^18^F-fluorodexoyglucose positron emission tomography (FDG-PET) is utilized for predicting tumor behavior molecularly and clinically, because this imaging modality accurately reflects tumor biology [1,2,3,4]. In breast cancer (BRC), numerous investigations have shown the contribution of tumor biology to increased glucose uptake in FDG-PET [5,6] and have demonstrated that the degree of glucose uptake is associated with aggressive tumor characteristics [7,8]. The standardized uptake value (SUV), which represents the degree of glucose uptake in FDG-PET, has been recognized as a prognostic and predictive factor.

Recent advances in molecular biology support the importance of glucose metabolism in malignancies [9]. In terms of glucose uptake, FDG-PET is a useful imaging tool for reflecting glucose metabolism [1]. Therefore, understanding glycolysis associated with FDG-PET in malignancy may offer novel therapeutic options that target metabolism in BRC. Previous investigations also showed a significant association between the SUV and prognostic indicators such as estrogen receptor (ER), progesterone receptor (PR), human epidermal growth factor receptor-2 (HER2), or histologic grade in BRC [1,4,5,10,11], indicating a close relationship between tumor aggressiveness and FDG-PET. Despite the metabolic or oncogenic significance of FDG-PET imaging, its characteristics have not been fully explored at the integrative genomics level, which might be because of the complex effects of glucose metabolism on multiple cancer progression steps. Therefore, there is a crucial need to address this issue and to identify key biomarkers that can be utilized in clinical practice.

For effective cancer therapy, it is important to classify patients precisely by combining molecular imaging and genomic techniques. Here, we identified a genomic signature associated with the SUV derived from FDG-PET in BRC. Using multiple BRC cohorts, we found the prognostic or therapeutic relevance of the signature. Integrated exploration of SUV, mutational, and gene expression alterations revealed that patients with a high SUV were responsive to immune checkpoint inhibitor (ICI) treatment, whose predictive variants were also discovered. Using experimental assays with BRC cell lines at the transcriptional and protein levels, we also verified a strong interaction between oncogenic transcription factors and metabolic indicators that were associated with SUV activity.

## 2. Results

### 2.1. Transcriptomic Profiling Reflecting SUV Alterations in BRC

Table 1 details the baseline characteristics of the 3710 BRC patients included in this study. Among the six patient cohorts, we first performed gene expression profiling in the Yonsei University (YSU) cohort containing 301 BRC patients, including 66 patients with BRC for whom SUV data were available. To identify a gene expression signature responsible for the SUV, we selected a gene set correlated with the SUV (Pearson correlation test; *p* < 0.01; |*r*| > 0.35). Hierarchical clustering analysis with 1424 genes divided the BRC patients into two subgroups according to the SUV: an SUV-high cluster (SHC) and an SUV-low cluster (SLC) (Figure S1A). When comparing sample clusters divided by SUV-correlated genes and subgroups dichotomized by an SUV cut-off (SUV of ≤ 4 or > 4), as used in our previous studies [1,4], we observed that two criteria demonstrated significantly similar BRC patient classifications (Fisher’s exact test; *p* = 1.71 × 10^−4^; Figure S1A). No difference between recurrences estimated by these two criteria was observed (Figure S1B), indicating that SUV-correlated genes reflect the SUV’s prognostic characteristic.

By exploring the expression pattern of 1424 genes, we intuitively found a distinct subset of genes corresponding to the SUV sample clusters (131 genes surrounding the dashed line in Figure S1A). These genes were more highly expressed in the SHC than in the SLC. Among them, cell-cycle-, cytokinesis-, or DNA-repair-associated genes, such as *TOP2A*, *AURKB*, *FOXM1*, and *BIRC5*, were observed, implying that SUV-associated subgroups are mediated by these genes. To validate SUV-associated gene expression patterns, we applied 1424 genes to other independent gene expression data in the YSU triple-negative breast cancer (TNBC) cohort (*n* = 84; Table 1), in which SUV data are also available. A hierarchical cluster analysis revealed significantly similar TNBC patient classifications between SUV and SUV-correlated genes, in which DNA-repair-associated genes were highly enriched in the SHC (Figure S1C), consistent with the results in the YSU cohort.

To identify a small gene set that still retains a discriminative power stratifying BRC patients into the SLC or SHC, we compared two gene sets correlated with the SUV in the YSU and YSU-TNBC cohorts (Pearson correlation tests; *p* < 0.01; |*r*| > 0.35). By intersecting two gene sets, we observed that 229 genes were common (Figure S2A). Hierarchical clustering analyses with these genes well stratified BRC patients into SUV-high or SUV-low subgroups in the two cohorts, in which DNA-repair-associated genes were still highly enriched in the SHC (Figure S2B,C), suggesting a set of the 229 genes that could be adopted into a diagnostic panel for classifying BRC patients into different risk subgroups (Table S1).

**Figure 1:**
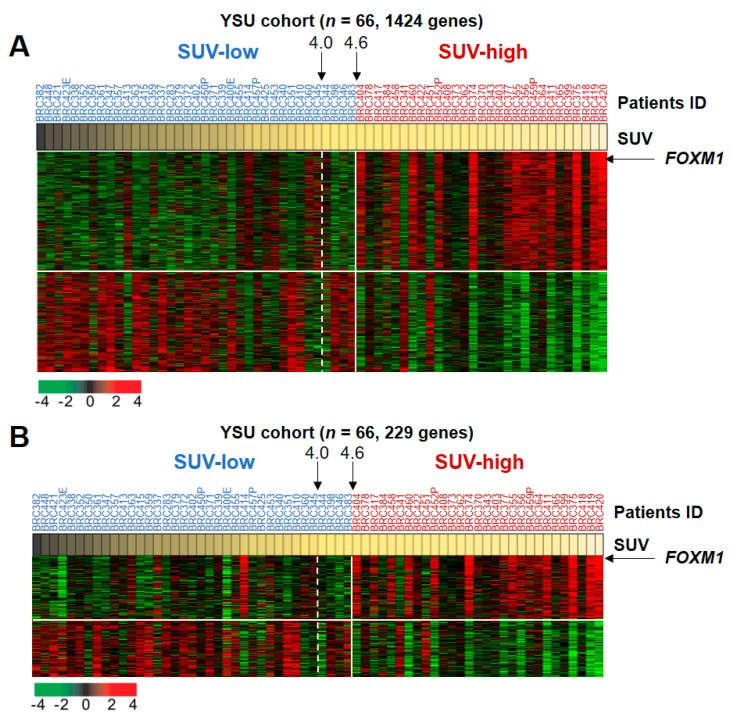

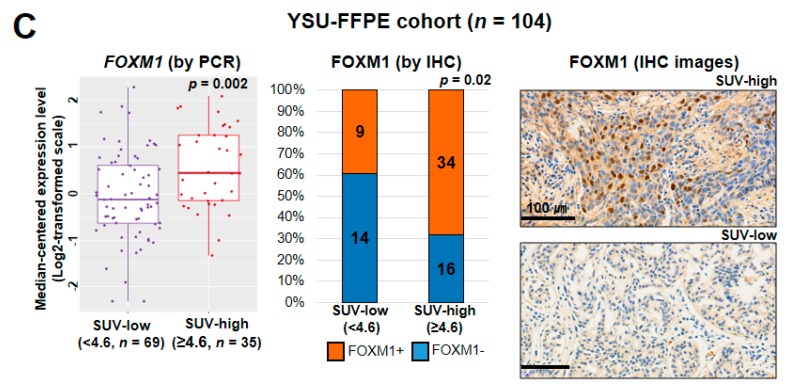
Comparison of the standard uptake value (SUV) and the expression patterns of SUV-correlated genes in the multiple breast cancer (BRC) patient cohorts. (**A**) Comparison of the SUV and the gene expression patterns in the YSU cohort. A total of 1424 genes were applied to generate a heat map of gene expression from 66 BRC patients whose SUV data were available. Genes were sorted by Pearson correlation coefficient between the gene expression level and the SUV. BRC samples were also sorted by the SUV. (**B**) Comparison of the SUV and the expression patterns of 229 genes in the YSU cohort. The 229 genes were commonly correlated with the SUV, both in the YSU and YSU-TNBC cohorts. (**C**) Examination of *FOXM1* mRNA expression and protein levels using formalin-fixed paraffin-embedded (FFPE) tissues in the YSU-FFPE cohort (*n* = 104). BRC patients were divided into two groups by an SUV cut-off of 4.6. The *p*-value in comparison of mRNA expression was obtained by a two-sample *t*-Test, whereas the *p*-value in comparison with protein levels was obtained by Fisher’s exact test. IHC, immunohistochemistry.

To date, the SUV cut-off for BRC patient classification has not been standardized. Because the SUV-correlated genes reflected a prognostic characteristic of the SUV, we sought to identify a threshold of the SUV that corresponded to the gene expression alterations. When BRC patients were heuristically stratified by expression patterns of 1424 SUV-correlated genes, we observed that the corresponding SUV cut-off was 4.6 (Figure 1A). When 229 SUV-correlated genes common in both YSU and YSU-TNBC cohorts were applied, the empirical stratification of patients with BRC still showed a corresponding SUV cut-off of 4.6 (Figure 1B), which might be a potential criterion of the SUV for classification of high-risk BRC patients. These results indicate that, although the cut-off was arbitrarily selected, high-risk BRC patients may be classified by a diagnostic panel consisting of several genes beyond SUV.

Among DNA-repair-associated genes, *FOXM1* was the top discriminator of the two risk subgroups as its mRNA expression levels were strongly correlated with the SUV (Pearson correlation test; *p* = 1.44 × 10^−6^; *r* = 0.55). To validate this observation, we also estimated mRNA and protein expression levels of FOXM1 in another independent cohort of formalin-fixed paraffin-embedded (FFPE) samples (*n* = 104, the YSU-FFPE cohort; Table 1), whose SUV data were also available. These additional assessments confirmed higher expression and nuclear staining patterns of FOXM1 in SUV-high rather than in SUV-low patients (Figure 1C), suggesting FOXM1 as a key factor to predict SUV activity.

### 2.2. Biological Insight into the SUV-Associated Genes

To explore the molecular characteristics compatible with the SUV, functional enrichment analysis of 1424 genes reflecting SUV alteration (Figure 1A) was carried out. The analysis results revealed that genes involved in cancer, cell cycle, and tissue development were strongly enriched. Consistent with the previous result (Figure S1A), a significant number of genes were revealed to be involved in DNA replication, recombination, and repair (Figure S3A), indicating that abnormal activity of the DNA repair system might markedly induce the poor prognosis of patients with BRC in the SHC.

To identify predominant regulators associated with SUV subgroups, an upstream regulator analysis of SUV-associated genes was performed. Examination of the enriched genes displayed important activated or inhibited regulators, the strongest overrepresentation of which was the predominant activation of *FOXM1* (Table S2). Among the significant upstream regulator candidates, we sought to identify downstream partners regulated by *FOXM1*, revealing a *FOXM1*–*AURKB*–*CDKN1A* signaling axis associated with the SUV subgroups (Figure S3B). *FOXM1* formed the primary hub of the gene network that was subsequently interconnected with other gene network hubs centered on *AURKB* and *CDKN1A*. The expression levels of *FOXM1* and *AURKB* were significantly higher in the SHC than in the SLC in the YSU cohort (two-sample *t*-test; each *p* < 0.001; Figure S3C), indicating that *FOXM1*–*AURKB* signaling may be a key genetic determinant associated with poor prognosis of BRC in the SHC patients. Although a gene network regulated by *CDKN1A* was significantly associated with the SUV subclusters, no significance between *CDKN1A* expression and the sample clusters was observed, indicating a contribution of *CDKN1A* not by gene expression changes, but by other biological events in the high-risk SHC patients.

We also performed regulator effects analysis via the Ingenuity Pathway Analysis tool (QIAGEN) to assess which biofunctions were significantly activated by upstream regulators with their effectors. SUV-associated regulators revealed the top regulator effects, activating cytokinesis and dysregulation of several cell cycle phases in tumor cells (Figure S3D). In the upper layer of the network, *FOXM1*, *CDKN1A*, and *MYC* [5], along with other important regulators, regulated many genes in the middle layer, such as *CDC20*, *AURKB*, *TOP2A*, *BIRC5*, and *CCNE1*, which are involved in the cell cycle, cytokinesis, or DNA repair, consistent with our previous findings (Figure S1A). The expression levels of these genes were significantly higher in the SHC than in the SLC in the YSU cohort (two-sample *t*-Test; each *p* < 0.001; Figure S3C), indicating a robust property of the SUV compatible with a biological mechanism by which *FOXM1*, along with other important regulators, synergistically activates downstream effectors, which subsequently leads to the dysregulation of cytokinesis or the cell cycle.

### 2.3. Prognostic Utility of the SUV Signature

**Figure 2:**
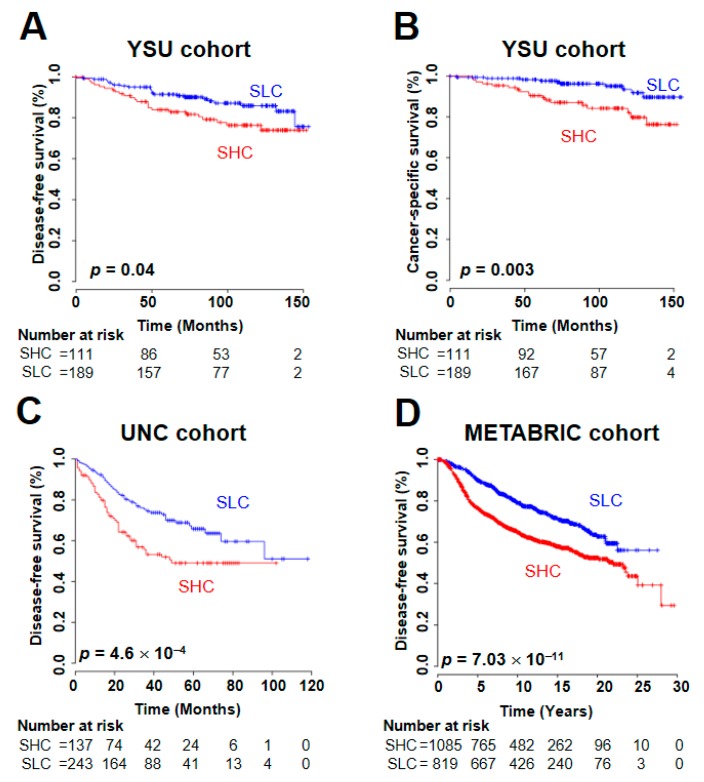
Prognosis of two subgroups dichotomized by the standardized uptake value (SUV) signature in the multiple breast cancer (BRC) patient cohorts. By a hierarchical clustering analysis of the SUV-associated genes, the BRC samples were divided into two groups: an SUV-high cluster (SHC) and an SUV-low cluster (SLC) (Figure S4). The survival rate of BRC patients in each subgroup was estimated. (**A**) In the YSU cohort, the disease-free survival (DFS) rate in the SHC was significantly lower than in the SLC (log-rank test; *p* = 0.043). (**B**) The cancer-specific survival rate in the SHC was also significantly lower than that in the SLC in the YSU cohort (log-rank test; *p* = 0.003). (**C**) When patients in the UNC cohort were divided into the SHC and SLC by hierarchical clustering using genes in the SUV signature, the DFS rate in the SHC was significantly lower than that in the SLC (log-rank test; *p* = 4.6 × 10^−4^). (**D**) When the same procedure was applied to the METABRIC cohort, the SUV signature showed statistical significance for predicting BRC DFS (log-rank test; *p* = 7.03 × 10^−11^).

The SUV clearly has prognostic significance [1]. We next sought to identify a molecular signature and to use the signature to verify its prognostic relevance in BRC. Gene expression data from all 301 samples were used to estimate prognosis in the YSU cohort. Hierarchical clustering analysis of the SUV-associated genes divided BRC patients into SLC and SHC. The disease-free survival (DFS), cancer-specific survival, and overall survival (OS) rates in the SHC were significantly lower than those in the SLC (Figure S4A and Figure 2A,B). We also validated the prognostic value of the signature in the University of North Carolina (UNC) [12], The Cancer Genome Atlas (TCGA) [13], and the Molecular Taxonomy of Breast Cancer International Consortium (METABRIC) [14] cohorts, in which the SUV signature was a robust predictor of BRC DFS and OS (Figure S4B–D and Figure 2C,D), consistent with a previous description of the SUV’s prognostic characteristic [15].

To assess the prognostic independence of the SUV signature, we combined clinical data from the YSU and UNC cohorts, in which the signature showed significant predictive value for BRC recurrence, and we applied Cox regression analyses to our signature with known clinicopathological risk factors. In the univariate and multivariate analyses, the SUV signature showed its statistical significance for recurrence prediction in BRC (hazard ratio [HR], 1.48; 95% confidence interval [CI], 1.05 to 2.09; *p* = 0.02; Table 2). Additional analyses in the METABRIC cohort also demonstrated the signature’s robustness in predicting BRC DFS (HR, 1.27; 95% CI, 1.12 to 1.45; *p* = 2.23 × 10^−4^; Table 2), indicating the independent clinical utility of the SUV signature.

### 2.4. Mutational Landscape of SUV Genomic Subtypes in BRC

**Figure 3:**
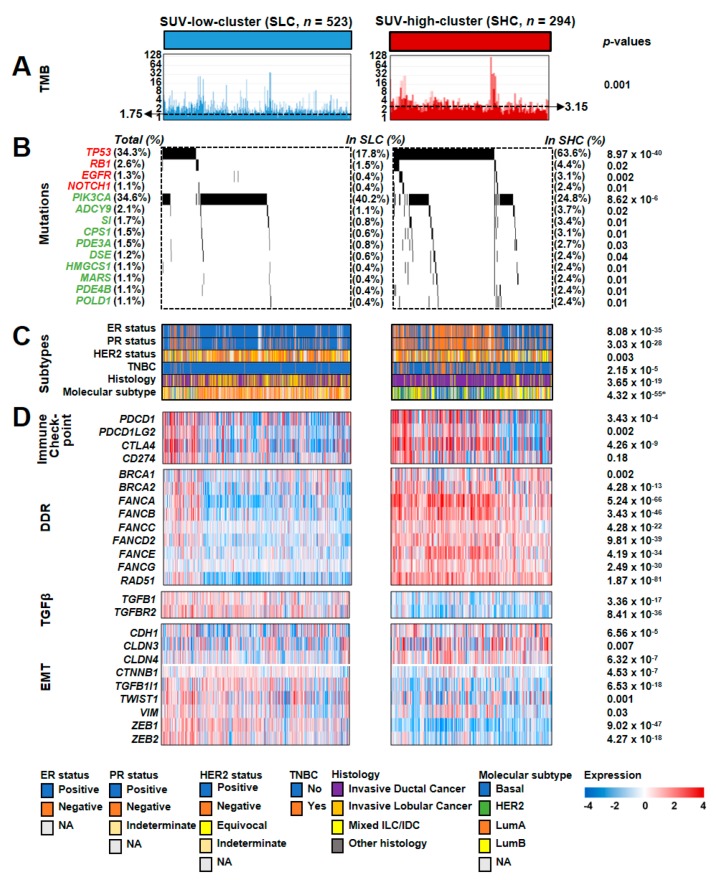
Association between standardized uptake value (SUV) subgroups and core molecular features in breast cancer (BRC). Molecular characteristics of the SUV subgroups were categorized by tumor mutation burden (TMB) (**A**), mutations (**B**), known subtypes (**C**), and heat maps of gene expression involved in core pathways (**D**). BRC samples were ordered by the overall mutation frequency of the genes involved in the oncogenic signature or metabolic pathways. In the panel of mutations, gene symbols with a red color indicate involvement in the oncogenic signature, while gene symbols with a green color indicate involvement in the metabolic pathways. The *p*-values in the TMB and gene expression categories were obtained by two-sample *t*-tests. The *p*-value in the molecular subtype category was obtained by the *χ*^2^ test, whereas the remaining *p*-values in the mutation or subtype categories were obtained by Fisher’s exact tests. DDR, DNA damage response; EMT, epithelial–mesenchymal transition.

Based on the transcriptomic and prognostic properties of the SUV signature, we sought to identify somatic alterations associated with the signature in the TCGA cohort. When the tumor mutation burden (TMB) was estimated, the TMBs in the SHC were significantly higher than those in the SLC (two-sample *t*-Test; *p* = 0.001; Figure 3A). When the mutation frequencies of the genes in the oncogenic signature or metabolic pathways were compared between the SLC and SHC, seven genes in the oncogenic signature and 54 genes in the metabolic pathways showed significantly different mutation frequencies (Figure S5A). We selected genes with a total mutation frequency of >1% across all BRC patients in the TCGA cohort and found that four genes in the oncogenic signature and 10 genes in the metabolic pathways had significantly different mutation rates between the SHC and SLC (Figure 3B). Among them, *TP53* was the top discriminator of the SUV subgroups. The total mutation rate of *TP53* was 34.3%, and the mutation frequency of *TP53* in the SHC was significantly higher than that in the SLC (Fisher’s exact test; *p* = 8.97 × 10^−40^). *PIK3CA* was another significant discriminator of the SUV subgroups. The total mutation rate of *PIK3CA* was 34.6%, and the mutation frequency of *PIK3CA* in the SHC was significantly lower than that in the SLC (Fisher’s exact test; *p* = 8.62 × 10^−6^), demonstrating a contrasting pattern of mutation frequency to that of *TP53* (Figure 3B). When comparing the mutation patterns between the SLC and the SHC in the METABRIC cohort, we also verified mutually exclusive mutation patterns of *TP53* and *PIK3CA* between the subtypes (Figure S5B), consistent with the TCGA cohort results.

We also compared known molecular and histological subtypes with somatic alterations illustrated by the SUV signature (Figure 3C). Regarding the ER and PR status, we observed significantly higher frequencies of negative BRC patients in the SHC (Fisher’s exact tests; *p* = 8.08 × 10^−35^ and *p* = 3.03 × 10^−28^, respectively). The frequencies of ER- or PR-negative status were higher among patients with mutated *TP53* but wild-type *PIK3CA* than among other patients, regardless of SUV subgroup. Regarding the HER2 status, moderate statistical significance was found for the difference between SUV subgroups (Figure 3C), for which we performed a signature-based re-stratification of BRC patients according to HER2 status. We divided the patients into the subgroups based on the HER2 status (i.e., negative, equivocal, or positive) and classified them based on the signature in each HER2 subgroup. The BRC patients in each HER2 subgroup were clearly classified into the SLC or the SHC, in which mutually exclusive *TP53* and *PIK3CA* mutation patterns and significant enrichment of ER-negative status in the SHC were also observed (Figure S6A). These results underscore the independent utility of the signature, regardless of HER2 status. The frequency of patients with TNBC in the SHC was profoundly higher than that in the SLC (Fisher’s exact test; *p* = 2.15 × 10^−5^), accounting for the poor prognosis of patients in the SHC. Importantly, the vast majority of TNBC patients were in the subgroup of patients with mutated *TP53* and wild-type *PIK3CA* (73.2%, 60 of 82 patients with TNBC; Figure 3C). When a signature-based stratification was applied to only TNBC patients (*n* = 299) in the METABRIC cohort, the SUV signature retained the mutually exclusive mutation patterns of *TP53* and *PIK3CA* between the SHC and the SLC (Figure S6B,C), suggesting the independent utility of the signature in TNBC. The majority of patients with invasive lobular cancer were in the SLC, while most patients in the SHC had invasive ductal cancer in the TCGA cohort (Fisher’s exact test; *p* = 3.65 × 10^−19^; Figure 3C). Most BRC patients with LumA were classified into the SLC, whereas many BRC patients with the basal subtype were classified into the SHC (*χ*^2^ test; *p* = 4.32 × 10^−55^; Figure 3C).

Based on the significant relationship between FOXM1 and the SUV activity in the previous results (Figure 1C and Figure S3), we further sought to identify an association between FOXM1 protein expression and known important molecular subtypes (LumA, LumB, HER2-negative, and TNBC). When estimating FOXM1 protein expression generated by reverse phase protein arrays (RPPA) in the TCGA cohort, we observed significantly different expression levels of FOXM1 across molecular subtypes (Figure S6D), indicating that FOXM1 may be a good discriminator well reflecting distinct molecular characteristics of the known subtypes.

### 2.5. Association Between the SUV Signature and the Response to Immune Checkpoint Inhibitors (ICIs)

The TMB has been shown to be a useful biomarker for predicting the response to ICIs [16,17]. With a significant difference in the TMB between the SUV subgroups (Figure 3A), we attempted to identify the predictive value of the SUV signature for ICI treatment response (Figure 3D). When the expression levels of immune checkpoint genes were investigated, we found significant differences in the expression between the SUV subgroups, except for *PD-L1* (*CD274*). Since the activation of DNA damage response and repair (DDR) genes was significantly associated with the ICI response [18], we also estimated the expression levels of DDR genes, demonstrating that the vast majority of DDR genes were significantly activated in the SHC. Interestingly, many BRC patients with mutant *TP53* and wild-type *PIK3CA* showed overall patterns of increased DDR gene expression, implying the predictive value of mutually exclusive *TP53* and *PIK3CA* mutations for predicting ICI responsiveness. TGFβ pathway genes were significantly downregulated in the SHC, and epithelial–mesenchymal transition (EMT) genes were differentially expressed between the SUV subgroups, consistent with the previous report that TGFβ attenuates the response to ICI [19]. Due to the non-availability of ICI responsiveness data at the genome level, despite clinical trials for ICI in BRC [20], we alternatively sought to validate the predictive value of the SUV signature for ICI (atezolizumab, a PD-L1 inhibitor) treatment in urothelial cancer (UC) [19]. When applying the SUV signature for BRC to the UC cohort, the rate of ICI responsiveness in the SHC was significantly higher than that in the SLC (Figure S7), indicating that ICI treatment is a possible option for patients classified into the SHC. Taken together, our findings suggest that gene expression-based SUV subgroups may reflect ICI responsiveness.

### 2.6. Experimental Characterization of the SUV Signature Mediated by FOXM1–LDHA Signaling

By observing several genes in the oncogenic or metabolic pathways associated with the SUV signature (Figure 3), we sought to identify upstream factors regulating *FOXM1* and its downstream effectors (Figure S3B). Re-examination of significantly mutated genes and the SUV signature showed a strongly overrepresented upstream network of *TP53* (Figure S8A). *TP53* formed a primary gene network hub that was subsequently interconnected with another gene network hub centered on *FOXM1*. Many genes in the metabolic pathways (i.e., *LDHA*, *PTEN*, *GART*, *PDE4B*, and *POLD1*) were regulated by *TP53*, among which *FOXM1* commonly regulated *LDHA*, which is involved in glycolysis–gluconeogenesis. The expression levels of glycolysis–gluconeogenesis genes were significantly differentiated between the SUV-associated subgroups (two-sample *t*-tests; each *p* < 0.05; Figure S8B–I). In common with the FOXM1 expression (Figure 1C), additional assessments of protein levels using FFPE tissues in the YSU-FFPE cohort who underwent preoperative FDG-PET confirmed higher pLDHA cytoplasmic staining in SUV-high than in the SUV-low patients (Figure S8J,K). These results suggest an additional property of the SUV to reflect glucose metabolism regulated by *TP53* and *FOXM1*.

**Figure 4:**
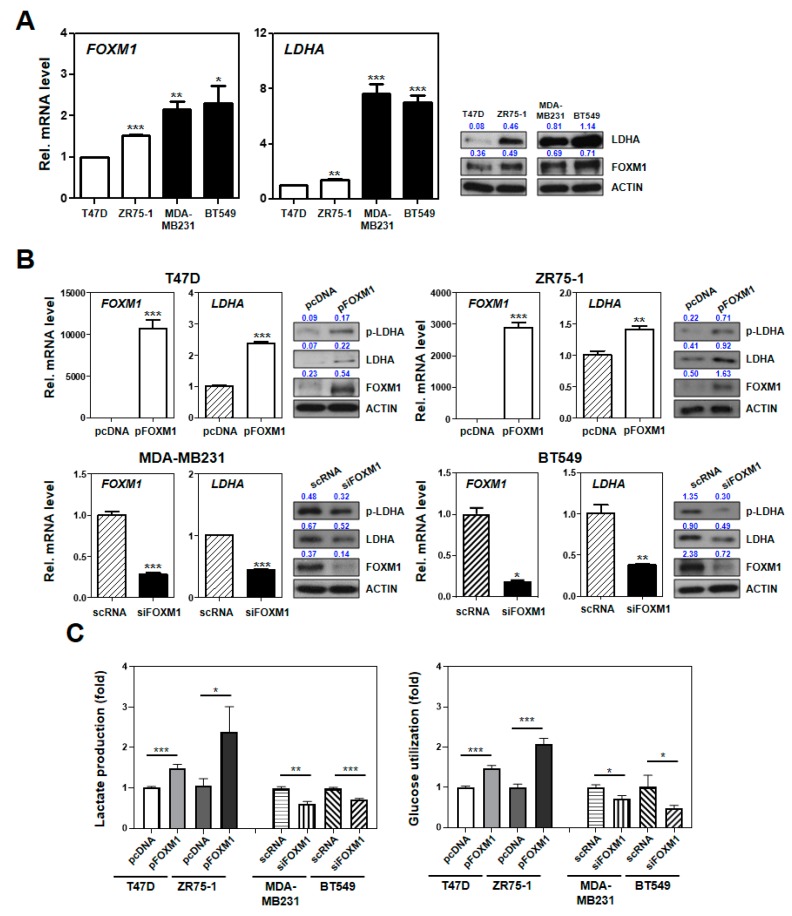
FOXM1 regulates LDHA expression, glucose utilization, and lactate production in breast cancer (BRC) cells. (**A**) Comparison between the expression levels of *FOXM1* and *LDHA* in cancer cells defined as SUV-high cluster (SHC; MDA-MB231 and BT549) and SUV-low cluster (SLC; T47D and ZR75-1). The left two panels show the mRNA levels of *FOXM1* and *LDHA* as determined by quantitative polymerase chain reaction (qPCR), and the right two panels show the protein expression levels of these two genes as determined by Western blotting. (**B**) In the upper panels, the SLC cell lines (T47D and ZR75-1) were transfected with pcDNA (control) or the FOXM1 overexpression vector (pFOXM1). In the bottom panels, the SHC cell lines (MDA-MB231 and BT549) were transfected with scrambled RNA (scRNA) or siFOXM1. The mRNA levels of *FOXM1* and *LDHA* were measured by quantitative reverse transcription (qRT)-PCR. (**C**) The SLC and SHC cells were cultured for 24 h. A lactate assay kit was used for LDH production analysis, and a colorimetric glucose assay kit was used for glucose utilization confirmation. The absorbance was measured at 330 mm. Each bar represents the mean ± standard deviation of three independent experiments; *p*-values were obtained by two-sample *t*-tests. * *p* < 0.05; ** *p* < 0.01; *** *p* < 0.001.

In vitro assays using *FOXM1* and *LDHA* were also carried out to verify the SUV characteristics to support glucose metabolism regulated by *FOXM1*. By applying the SUV signature into 52 BRC cell lines, we characterized two BRC cell groups for further studies (Figure S9A). Through further estimation of the mRNA and protein levels of FOXM1 and LDHA, we selected MDA-MB231 and BT549 as SHC cell lines and T47D and ZR75-1 as SLC cell lines (Figure 4A, Figure S9B,C, Figure S10, Figure S11).

When the two SLC cell lines were transfected with pFOXM1, the mRNA and protein levels of FOXM1, LDHA, and the phosphorylation of LDHA were increased. However, we transfected the two SHC cell lines with siFOXM1 and confirmed a decrease in the levels of LDHA and pLDHA (Figure 4B and Figure S12). We also performed a lactate and glucose uptake assay to investigate whether FOXM1 expression is associated with lactate production, LDHA activity, and glucose utilization in BRC cells. From these data, we found that FOXM1 overexpression increased glucose utilization and lactate production in SLC cells. In contrast, the levels of glucose utilization and lactate production were decreased in siFOXM1-transfected SHC cells (Figure 4C). These results indicate that FOXM1 transcriptionally regulates the expression of LDHA in BRC, suggesting that the aggressiveness in the SHC may be modulated by activation of the FOXM1–LDHA signaling pathway.

## 3. Discussion

The SUV is clinically relevant in BRC prognosis [1]. To uncover the molecular activities in the FDG-PET process in BRC patients, we analyzed gene expression and mutation data and identified a signature reflecting the best characteristics of the SUV in multiple BRC cohorts. The prognostic utility of the SUV signature was robust and validated independently. In terms of mutational patterns, the signature was significantly associated with mutually exclusive *TP53* and *PIK3CA* mutations. We also provided clinical evidence that high-risk patients identified by the signature might have a higher responsiveness to ICI treatment compared to those with low risk. Additionally, a putative signaling pathway defined by *TP53–FOXM1–LDHA* might be responsible for the molecular activity determining the SUV.

On the basis of increased aerobic glycolysis accompanied by glucose uptake in malignant tumors, FDG-PET is clinically utilized and enables clinicians to measure the degree of glucose uptake by cancer cells. The SUV, an objective indicator of glucose uptake, is obtained by FDG-PET scanning and aids the understanding of tumor biology with respect to glucose metabolism. Based on the robust diagnostic relevance of FDG-PET imaging, the SUV has been applied in various clinical fields, including molecular PET application such as ^89^Zr-atezolizumab imaging for predicting the response to ICI-based immunotherapy [21]. Despite the usefulness of FDG-PET, its characteristics have been poorly surveyed at the integrative genomics level. Considering that our previous works indicated the clinical value of the SUV in BRC [1,22], we performed gene expression profiling in BRC patients who underwent FDG-PET. By multistep investigation, we verified the predictive values of the SUV signature, especially for ICI treatment response, and the responsible signaling pathway consisting of *TP53*, *FOXM1*, and *LDHA*. We propose our genomic signature as a basic resource describing the molecular activity associated with the SUV derived from FDG-PET scanning.

The current study revealed several molecular features and a credible signaling pathway responsible for SUV-associated activity. Mutation profiling revealed that *TP53* and *PIK3CA*, previously well-known factors explaining BRC heterogeneity [23,24,25,26], were the molecules most significantly distinguished between the SUV-associated subgroups, suggesting a possible diagnostic method using *TP53* and *PIK3CA* mutations to support FDG-PET scan performance. The high TMB and distinct activity of DDR and EMT genes in the SHC subgroup also support the significant value of the SUV signature as a predictive signature for ICI treatment [16,17,18,19]. The signature-based re-stratifications of BRC patients demonstrated the independent utility of the SUV signature regardless of HER2 or TNBC status, proposing an aggressive treatment option including ICI-based immunotherapy for the SHC patients with equivocal molecular status such as HER2-equivocal. Additionally, gene network analysis revealed that a regulatory relationship between *TP53* and *FOXM1* was associated with different SUV-associated activities. Since the oncogenic transcription factor *FOXM1* is negatively regulated by the tumor suppressor *TP53* [27], aggressiveness in the SHC may be crucially mediated by *TP53*. We also observed that *TP53* and *FOXM1* regulated *LDHA* cooperatively. FOXM1 is reported to be associated with tumor aggressiveness and Warburg effect regulation in several cancers [28,29,30]. Elevated FOXM1–LDHA signaling increased cancer cell growth and metastasis [30,31]. In this study, we characterized the roles of FOXM1 in the Warburg effect in terms of LDH activity, glucose utilization, and lactate production via the regulation of the expression of LDHA, the key enzyme in aerobic glycolysis. FOXM1 positively regulates aerobic glycolysis and aggressiveness in BRC, suggesting that FOXM1–LDHA signaling has promising new clinical implications for diagnostic and therapeutic strategies.

In conclusion, we identified a genomic signature revealing distinct molecular subtypes of BRC associated with the SUV obtained from FDG-PET scanning. We also evaluated two clinical aspects of the signature—prognosis and ICI responsiveness. Our results uncover genomic and metabolomic characteristics of glucose uptake captured by FDG-PET, supporting an understanding of glucose metabolism, as well as the poor prognosis of BRC patients with a high SUV. Although our data reveal a significance of the SUV signature, a further validation study is warranted to identify an easily assessable biomarker consisting of a small gene set that still robustly retains the SUV activity.

## 4. Materials and Methods

### 4.1. Patients and Tissue Samples

We prospectively collected tumor tissues from specimens of surgically resected BRC at the Gangnam Severance Hospital, Yonsei University College of Medicine, Seoul, Korea, between July 1997 and December 2007. After applying the exclusion criteria, 301 patients with invasive BRC were ultimately enrolled for gene expression profiling (*n* = 301, the YSU cohort). The following patients were excluded: patients with pure in situ carcinoma of the breast; recurrent or metastatic disease; bilateral BRC; or BRC of nonepithelial origin, such as phyllodes tumor, sarcoma, or lymphoma; as well as those receiving neoadjuvant chemotherapy. Among the 301 patients with BRC, the SUV data of 66 patients who underwent preoperative FDG-PET were available. We also collected tumor tissues from specimens of surgically resected TNBC at the Gangnam Severance Hospital, Yonsei University College of Medicine, Seoul, Korea (*n* = 84, the YSU-TNBC cohort). These TNBC samples were independently collected with the YSU cohort, and their SUV data were also available. For further validation, we also collected FFPE tissues in other independent BRC patient cohort who underwent preoperative FDG-PET (*n* = 104, the YSU-FFPE cohort). Our study was approved by the institutional review board (IRB) of Gangnam Severance Hospital, Yonsei University, Seoul, Korea (ethic code: 3-2013-0136), in accordance with the good clinical practice guidelines under the Declaration of Helsinki. The IRB granted a waiver of written documentation of informed consent from all participants because of the retrospective study design. Details of the three BRC patient cohorts with SUV data availability are described in Table 1.

### 4.2. 18F-Fluorodexoyglucose Positron Emission Tomography (FDG-PET)

The procedure for FDG-PET was previously reported [22]. The SUV was calculated by measuring ^18^F-fluorodexoyglucose uptake by the primary tumor in the region of interest, as follows: SUV = [maximal radioactivity concentration in the region of interest]/[injected dose/patient weight (kg)].

### 4.3. RNA Extraction, Microarray Experiments, and Data Processing

Total RNA was isolated by TRIzol reagent (Life Technologies, Carlsbad, CA, USA) according to the manufacturer’s protocol. For gene expression profiling in the YSU cohort, 500 ng of total RNA was used for hybridization labeling according to the manufacturer’s protocols (Illumina HumanHT-12 expression BeadChip, version 3.0). Arrays were scanned with an Illumina Bead Array Reader confocal scanner (BeadStation 500GXDW; Illumina, Inc., San Diego, CA, USA) according to the manufacturer’s instructions. For gene expression profiling in the YSU-TNBC cohort, 2 μg of purified total RNA was used in the One-Cycle Eukaryotic Target Labeling Assay and hybridized to the array for 16 h at 45 °C (Affymetrix Human Genome U133 Plus 2.0 arrays). Arrays were scanned on an Affymetrix GeneChip 3000 7G scanner according to the manufacturer’s instructions.

After scanning, the gene expression data were normalized using quantile normalization in the R language environment (version 3.5.1). Measured gene expression values were log2 transformed and median centered across genes and samples.

### 4.4. Public Datasets of BRC Patients

Three independent cohorts of patients (*n* = 3221) with BRC were used for this study. The mRNA expression (RNA-Seq) data from The Cancer Genome Atlas database was obtained from the cBioPortal website (http://www.cbioportal.org; *n* = 817, the TCGA cohort) [13]. We downloaded the mRNA quantification data (generated by RSEM software), to which log2 transformation and quantile normalization were applied. To search for mutation profiles of BRC in the TCGA cohort, the variant data for genes in the oncogenic signature (64 genes) and metabolic pathways (1114 genes) were also collected from the cBioPortal. We additionally obtained a gene expression dataset of BRC patients from the University of North Carolina Lineberger Comprehensive Cancer Center (*n* = 500, the UNC cohort) [12], which is freely available in the National Center for Biotechnology Information (NCBI) Gene Expression Omnibus (GEO) database. Gene expression data for the UNC cohort were generated by combining data from four subpatient cohorts, including GSE18229, GSE20624, GSE2741, and GSE6128. We also collected mRNA expression data from a study of 1904 BRC patients conducted by the Molecular Taxonomy of Breast Cancer International Consortium (*n* = 1904, the METABRIC cohort) [14]. The variant data of the METABRIC cohort were also obtained from the cBioPortal. All genomic data used in this study contain patient survival and follow-up time data to estimate the prognostic relevance of the signatures. The baseline characteristics of the BRC patient cohorts whose datasets are publicly available are described in Table 1.

### 4.5. Statistical Analysis

To generate a gene set highly associated with the SUV score, we applied a Pearson correlation test to the gene expression data from the YSU cohort and selected genes with significant correlation coefficients with the SUV (|*r*| > 0.35 and *p* < 0.01). Using a gene expression data matrix consisting of a gene feature and its correlated genes, we performed hierarchical clustering analysis with the centered correlation coefficient as the measure of similarity, and used a complete linkage clustering method. According to the patient clustering results, the patients were divided into two subgroups. The Kaplan–Meier method was used to calculate the time to death or recurrence, and differences between the times were assessed using log-rank statistics. To estimate the independent utility of the signature, we performed a multivariate Cox regression analysis combined with known clinicopathological risk factors. To estimate the significance of gene expression differences between the patient subgroups, we performed a two-sample *t*-test for each gene.

Gene set enrichment analysis was carried out to identify the most significant gene sets associated with the disease process, molecular and cellular functions, and physiological and developmental conditions. The significance of overrepresented gene sets was estimated by Fisher’s exact test. To explore the relationships among the genes in the signature, we performed an upstream regulator analysis that searched known targets of each regulator in the dataset and compared their direction of change to the expected change based on previously published literature. Gene set enrichment and upstream regulator analyses were performed using Ingenuity Pathway Analysis (QIAGEN, Redwood City, CA, USA).

To estimate the TMB of each BRC patient in the TCGA cohort, the total number of somatic mutations was normalized to the exonic coverage of the reference genome (Assembly ID: GRCh38) in megabases. For mutation profiling of BRC patients, we obtained the oncogenic signature (64 genes) and metabolic pathway (1114 genes) gene sets from a molecular signature database (MSigDB, version 6.1). To obtain the genes in the oncogenic signature, we downloaded gene sets from the C6 category in the MSigDB collections. To obtain the genes in the metabolic pathways, we downloaded the Kyoto Encyclopedia of Genes and Genomes (KEGG) pathway gene sets from the C2 category in the MSigDB and selected the subgene sets associated with metabolism. The full list of metabolic processes with the included genes in this study is provided in Table S3.

### 4.6. Cell Culture

The human BRC cell lines BT549, BT20, T47D, ZR75-1, SKBR3, BT474, and MDA-MB453 were purchased from the Korean Cell Line Bank. Other cell lines (MDA-MB231 and MCF7) were obtained from the American Type Culture Collection (ATCC, Manassas, VA, USA). Cells from the ATCC were certified by the results of a short tandem repeat (STR) DNA profiling assay, a cytochrome C oxidase I assay, and a mycoplasma contamination assay. The nine BRC cell lines were characterized by the short tandem repeat (STR)-polymerase chain reaction (PCR) method and screened for mycoplasma contamination.

The BRC cells used in this study were cultured in Roswell Park Memorial Institute (RPMI)-1640 medium (HyClone, Logan, UT, USA) supplemented with 10% fetal bovine serum (FBS, HyClone) and 1% penicillin/streptomycin (HyClone) (hereafter referred to as complete RPMI1640 medium) at 37 °C in 5% CO_2_.

### 4.7. Transfection, Plasmids, and Small Interfering RNA (siRNA)

All cells were transfected using jetPRIME reagent (Cat. 114-15, Polyplus Transfection, Inc., NY, USA) at a ratio of DNA to reagent of 1:3 according to the manufacturer’s protocol. In our previous study, the pcDNA6-V5-His-tag expression vector was purchased from Invitrogen (Cat. V22020, Carlsbad, CA) and pFOXM1 was provided by Dr. Ju-Seog Lee (MD Anderson Cancer Centre, TX, USA) [32]. Scrambled RNA (scRNA) was purchased from GenePharma (Shanghai Co., Ltd., China), and siFOXM1 was synthesized at the ST Pharm Oligo center (ST Pharm, Seoul, Korea).

### 4.8. Quantitative Real-Time (qRT) PCR

The nine BRC cell lines were seeded in a six-well plate at a density of 2 × 10^5^ cells/well in complete RPMI1640 medium. Cells were transfected for 24 h, and total cellular RNA was isolated using RNAiso Plus (Cat. 9109, Takara Bio, Inc., Otsu, Japan). Total RNA (500 ng) was isolated, and cDNA was synthesized as described [32]. qRT-PCR was performed with TB Green Premix Ex Taq (Cat. RR420, Takara Bio, Inc., Otsu, Japan) on a Bio-Rad CFX96TM Real-time system and a C1000 TouchTM Thermal Cycler (Bio-Rad Laboratories, Inc., Hercules, CA, USA) under the following thermal cycling conditions: 95 °C for 15 s; 30 cycles at 94 °C for 30 s, 64 °C for 30 s, and 72 °C for 15 s; and 72 °C for 10 min. The qRT-PCR primer sequence sets are shown in Table S4.

### 4.9. Western Blotting and Antibodies

Cells were lysed with radioimmunoprecipitation assay (RIPA) buffer (Cat. IBS-BR002, iNtRON Biotechnology, Burlington, MA, USA) and the protein concentration was measured by the BSA method using a PierceTM BCA Protein Assay Kit (Cat. 23227, Thermo Scientific, Rockford, IL, USA). The antibodies used in immunoblotting were specific for FOXM1 (Cat. A301-532A, Bethyl Laboratories, Montgomery, TX, USA), LDHA (Cat. sc-137243, Santa Cruz Biotechnology, TX), and β-actin (Cat. 4967, Cell Signaling Technology, Inc., Danvers, MA, USA).

### 4.10. Lactate Production and Glucose Utilization Assay

Cells were transfected with plasmids and siRNAs and prepared for glucose utilization and lactate production assays. The lactate production assay was performed with a Lactate Assay kit according to the manufacturer’s protocol (Cat. K627-100). For the glucose utilization assay, transfected cells were incubated for 24 h. Then, the medium was replaced with phenol red-free RPMI supplemented with 1% FBS and 20 mmol/l oxamate sodium, and cells were maintained in continuous culture for 3 days. The glucose concentration in the medium was measured using a colorimetric glucose assay kit (Cat. K686-100, BioVision, Inc., Milpitas, CA, USA) and normalized according to cell number [31].

### 4.11. Data Availability

The gene expression datasets with SUV data are available in the NCBI GEO public database (https://www.ncbi.nlm.nih.gov/geo/) under the data series accession numbers GSE131769 and GSE135565.

## 5. Conclusions

In this study, a genomic signature exhibiting distinct molecular subtypes of BRC associated with the SUV obtained from FDG-PET scanning was identified. Two important clinical aspects of the signature, prognosis and ICI responsiveness, were also evaluated. We also found that a signaling axis defined by *TP53*–*FOXM1–LDHA* might be an important mediator in the FDG-PET process. Our results uncover genomic and metabolomic characteristics of glucose uptake captured by FDG-PET, supporting an understanding of glucose metabolism, as well as the poor prognosis of BRC patients with a high SUV.

## Figures and Tables

**Table 1:** Baseline characteristics of breast cancer patient cohorts.

Variables	YSU Cohort	YSU-TNBC Cohort	YSU-FFPE Cohort	UNC Cohort	TCGA Cohort	METABRIC Cohort
Patients (*n*)	301	84	104	500	817	1904
Age (years)						
Median	47	49	48	55	59	62
Range	22–78	34–77	28–72	24–93	26–90	22–91
Histology (%)						
Infiltrating Ductal Carcinoma	260 (86.38)	74 (88.1)	84 (80.8)		599 (73.3)	1454 (76.37)
Infiltrating Lobular Carcinoma	7 (2.33)	1 (1.2)	12 (11.5)		143 (17.5)	142 (7.46)
Mucinous Carcinoma	12 (3.99)	1 (1.2)	2 (1.9)		14 (1.7)	22 (1.16)
Medullary Carcinoma	5 (1.66)	2 (2.4)	0 (0)		5 (0.6)	25 (1.31)
Metaplastic Carcinoma	2 (0.66)	1 (1.2)	0 (0)		3 (0.4)	1 (0.05)
Others	15 (4.98)	4 (4.8)	6 (5.8)		54 (6.6)	245 (12.87)
NA		1 (1.2)		500 (100)		15 (0.79)
T classification						
T1	108 (35.88)	35 (41.7)	77 (74.0)		219 (26.8)	475 (24.95)
T2	166 (55.15)	46 (54.8)	26 (25.0)		459 (56.2)	800 (42.02)
T3	22 (7.31)	3 (3.6)	1 (1.0)		105 (12.9)	115 (6.04)
T4	0 (0)	0 (0)	0 (0)		34 (4.2)	9 (0.47)
NA	5 (1.66)			500 (100)		505 (26.52)
N classification						
N0	161 (53.49)	58 (69.0)	88 (84.6)	182 (36.4)	382 (46.8)	993 (52.15)
N1	83 (27.57)	21 (25.0)	15 (14.4)	189 (37.8)	278 (34)	604 (31.72)
N2	35 (11.63)	4 (4.8)		26 (5.2)	85 (10.4)	204 (10.71)
N3	22 (7.31)	1 (1.2)		0 (0)	58 (7.1)	103 (5.41)
NA			1 (1.0)	103 (20.6)	14 (1.7)	
AJCC stage						
I	77 (25.58)	29 (34.5)	70 (67.3)		140 (17.1)	470 (24.68)
II	157 (52.16)	50 (59.5)	33 (31.7)		461 (56.4)	681 (35.77)
III	62 (20.6)	5 (6.0)	1 (1.0)		184 (22.5)	280 (14.71)
IV	0 (0)	0 (0)	0 (0)		13 (1.6)	0 (0)
NA	5 (1.66)			500 (100)	19 (2.3)	473 (24.84)
ER						
Positive	188 (62.46)		102 (98.0)	243 (48.6)	593 (72.6)	1459 (76.63)
Negative	113 (37.54)	84 (100)	1 (1.0)	243 (48.6)	174 (21.3)	445 (23.37)
NA			1 (1.0)	14 (2.8)	50 (6.1)	
PR						
Positive	184 (61.13)		85 (81.7)		522 (63.9)	1009 (52.99)
Negative	117 (38.87)	84 (100)	18 (17.3)		251 (30.7)	895 (47.01)
NA			1 (1.0)	500 (100)	44 (5.4)	
HER2						
Positive	44 (14.62)		1 (1.0)	78 (15.6)	121 (14.8)	236 (12.39)
Negative	216 (71.76)	84 (100)	101 (97.1)	369 (73.8)	417 (51)	1668 (87.61)
NA	41 (13.62)		2 (1.9)	53 (10.6)	279 (34.1)	
SUV data availability						
Yes	66 (21.9)	84 (100)	104 (100)			
No	235 (78.1)			500 (100)	817 (100)	1904 (100)
Adjuvant chemotherapy						
Yes	270 (89.7)	83 (98.8)	27 (26.0)		40 (4.9)	396 (20.8)
No	18 (5.98)	1 (1.2)	76 (73.0)		12 (1.5)	1508 (79.2)
NA	13 (4.32)		1 (1.0)	500 (100)	765 (93.6)	
Recurrence, n	47	16	1	117	85	623
Death, n	35	5	1	91	120	1103
Median follow-up (month)	93.5	67.5	45.5	30.5	28.9	115.6

Abbreviations: YSU, Yonsei University; TNBC, triple-negative breast cancer; FFPE, formalin-fixed paraffin-embedded; UNC, University of North Carolina; TCGA, The Cancer Genome Atlas; METABRIC, Molecular Taxonomy of Breast Cancer International Consortium; NA, not available; ER, estrogen receptor; PR, progesterone receptor; HER2, human epidermal growth factor receptor-2.

**Table 2:** Univariate and multivariate Cox regression analysis of disease-free survival in breast cancer.

Variable		Univariate			Multivariate		
		*n*	HR (95% CI)	*p*-Value	*n*	HR (95% CI)	*p*-Value
Combined with YSU and UNC cohorts	Age (>35 years or not)	681	0.61 (0.38–0.98)	0.04	681	0.74 (0.45–1.25)	0.26
	Node status (Positive or Negative)	681	1.02 (0.65–1.6)	0.93		0.94 (0.58–1.5)	0.79
	ER status (Positive or Negative)	681	0.55 (0.4–0.75)	1.84 × 10^−4^		0.66 (0.47–0.94)	0.02
	HER2 status (Positive or Negative)	681	1.36 (0.95–1.94)	0.09		1.43 (0.99–2.08)	0.06
	SUV-signature (SHC vs. SLC *)	681	1.78 (1.31–2.42)	2.1 × 10^−4^		1.48 (1.05–2.09)	0.02
METABRIC cohort	Age (>35 years or not)	1904	0.62 (0.4–0.97)	0.04	1904	1.26 (0.81–1.95)	0.31
	Node status (Positive or Negative)	1904	2.26 (1.92–2.66)	7.07 × 10^−23^		1.68 (1.46–1.86)	2.88 × 10^−16^
	ER status (Positive or Negative)	1904	0.58 (0.49–0.69)	7.32 × 10^−10^		1.04 (0.89–1.21)	0.66
	HER2 status (Positive or Negative)	1904	2.05 (1.67–2.51)	3.57 × 10^−12^		1.37 (1.14–1.64)	8.27 × 10^−4^
	SUV-signature (SHC vs. SLC *)	1904	1.74 (1.47–2.05)	1.21 × 10^−10^		1.27 (1.12–1.45)	2.23 × 10^−4^

* Predicted outcome in Figure 2 was used for analysis. Abbreviations: HR, hazard ratio; CI, confidence interval; SHC, SUV-high cluster; SLC, SUV-low cluster.

## Supplementary Materials

The following are available online at https://www.mdpi.com/2072-6694/12/2/497/s1, Figure S1: Gene expression pattern of the standardized uptake value (SUV) associated genes in multiple breast cancer (BRC) patient cohorts. Figure S2: Gene expression patterns of the standardized uptake value (SUV) signature in multiple breast cancer (BRC) patient cohorts. Figure S3: Gene set enrichments and gene-to-gene network analyses of the 1424 genes with a change in expression that correlated with standardized uptake value (SUV). Figure S4: Gene expression patterns and overall survival of two subgroups dichotomized by the standardized uptake value (SUV) signature in the multiple breast cancer (BRC) patient cohorts. Figure S5: Mutation profile comparison between SUV-low cluster (SLC) and SUV-high cluster (SHC) in The Cancer Genome Atlas (TCGA; *n* = 817) and Molecular Taxonomy of Breast Cancer International Consortium (METABRIC; *n* = 1904) cohorts. Figure S6: Subset analyses of expression patterns of genes in the standardized uptake value (SUV) signature. Figure S7: Predictive value of the standardized uptake value (SUV) signature for predicting response to immune checkpoint inhibitors (ICIs) in metastatic urothelial cancers. Figure S8: Standardized uptake value (SUV)-associated gene networks and comparison of mRNA and protein expression levels. Figure S9: mRNA and protein expression patterns across human breast cancer (BRC) cell lines. Figure S10: Uncropped blots showing all the western bands in human breast cancer (BRC) cell lines. Figure S11: Uncropped blots showing all the western bands in four human breast cancer (BRC) cell lines (T47D, ZR65-1, MDA-MD231, and BT549). Figure S12: Whole blots showing all the western bands in four human breast cancer (BRC) cell lines (T47D, ZR65-1, MDA-MD231, and BT549). Table S1: 229 genes significantly correlated with the SUV. Table S2: Prediction of activated upstream regulators in the SHC. Table S3: Kyoto Encyclopedia of Genes and Genomes (KEGG) pathways associated with metabolism and their involved genes. Table S4: Primer sequences of the genes for qRT-PCR analysis.
