# Supplementary material for: Genomic Signature of the Standardized Uptake Value in 18F-Fluorodeoxyglucose Positron Emission Tomography in Breast Cancer
Source: Cancers (Basel). 2020 Feb 20;12(2):497. doi: 10.3390/cancers12020497 (PMC7072341; doi:10.3390/cancers12020497)
Supplement: Supplementary file 1 [file cancers-12-00497-s001.zip › Supplementary_figs.pptx]

## Slide 1
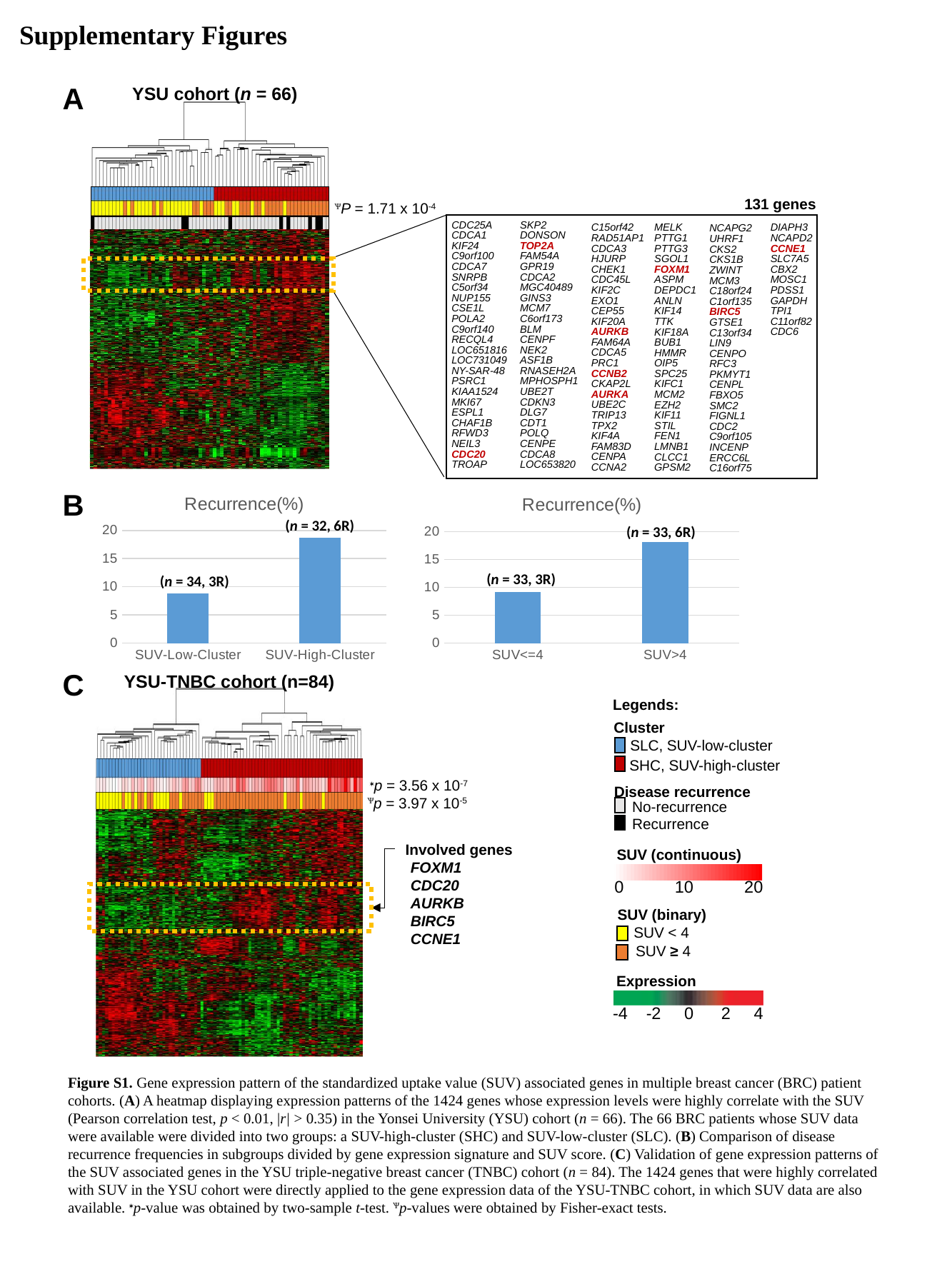

Supplementary Figures
A
YSU cohort (n = 66)
131 genes
ѰP = 1.71 x 10^-4^
CDC25A
CDCA1
KIF24
C9orf100
CDCA7
SNRPB
C5orf34
NUP155
CSE1L
POLA2
C9orf140
RECQL4
LOC651816
LOC731049
NY-SAR-48
PSRC1
KIAA1524
MKI67
ESPL1
CHAF1B
RFWD3
NEIL3
CDC20
TROAP
SKP2
DONSON
TOP2A
FAM54A
GPR19
CDCA2
MGC40489
GINS3
MCM7
C6orf173
BLM
CENPF
NEK2
ASF1B
RNASEH2A
MPHOSPH1
UBE2T
CDKN3
DLG7
CDT1
POLQ
CENPE
CDCA8
LOC653820
C15orf42
RAD51AP1
CDCA3
HJURP
CHEK1
CDC45L
KIF2C
EXO1
CEP55
KIF20A
AURKB
FAM64A
CDCA5
PRC1
CCNB2
CKAP2L
AURKA
UBE2C
TRIP13
TPX2
KIF4A
FAM83D
CENPA
CCNA2
DIAPH3
NCAPD2
CCNE1
SLC7A5
CBX2
MOSC1
PDSS1
GAPDH
TPI1
C11orf82
CDC6
MELK
PTTG1
PTTG3
SGOL1
FOXM1
ASPM
DEPDC1
ANLN
KIF14
TTK
KIF18A
BUB1
HMMR
OIP5
SPC25
KIFC1
MCM2
EZH2
KIF11
STIL
FEN1
LMNB1
CLCC1
GPSM2
NCAPG2
UHRF1
CKS2
CKS1B
ZWINT
MCM3
C18orf24
C1orf135
BIRC5
GTSE1
C13orf34
LIN9
CENPO
RFC3
PKMYT1
CENPL
FBXO5
SMC2
FIGNL1
CDC2
C9orf105
INCENP
ERCC6L
C16orf75
### Chart:
| Category | Recurrence(%) |
|---|---|
| SUV-Low-Cluster | 8.823529411764705 |
| SUV-High-Cluster | 18.75 |
### Chart:
| Category | Recurrence(%) |
|---|---|
| SUV<=4 | 9.09090909090909 |
| SUV>4 | 18.18181818181818 |B
(n = 32, 6R)
(n = 33, 6R)
(n = 33, 3R)
(n = 34, 3R)
C
YSU-TNBC cohort (n=84)
Legends:
Cluster
SLC, SUV-low-cluster
SHC, SUV-high-cluster
p = 3.56 x 10^-7^
Disease recurrence
Ѱp = 3.97 x 10^-5^
No-recurrence
Recurrence
Involved genes
SUV (continuous)
FOXM1
CDC20
AURKB
BIRC5
CCNE1
0 10 20
SUV (binary)
SUV < 4
SUV ≥ 4
Expression
-4 -2 0 2 4
Figure S1. Gene expression pattern of the standardized uptake value (SUV) associated genes in multiple breast cancer (BRC) patient cohorts. (A) A heatmap displaying expression patterns of the 1424 genes whose expression levels were highly correlate with the SUV (Pearson correlation test, p < 0.01, |r| > 0.35) in the Yonsei University (YSU) cohort (n = 66). The 66 BRC patients whose SUV data were available were divided into two groups: a SUV-high-cluster (SHC) and SUV-low-cluster (SLC). (B) Comparison of disease recurrence frequencies in subgroups divided by gene expression signature and SUV score. (C) Validation of gene expression patterns of the SUV associated genes in the YSU triple-negative breast cancer (TNBC) cohort (n = 84). The 1424 genes that were highly correlated with SUV in the YSU cohort were directly applied to the gene expression data of the YSU-TNBC cohort, in which SUV data are also available. p-value was obtained by two-sample t-test. Ѱp-values were obtained by Fisher-exact tests.

## Slide 2
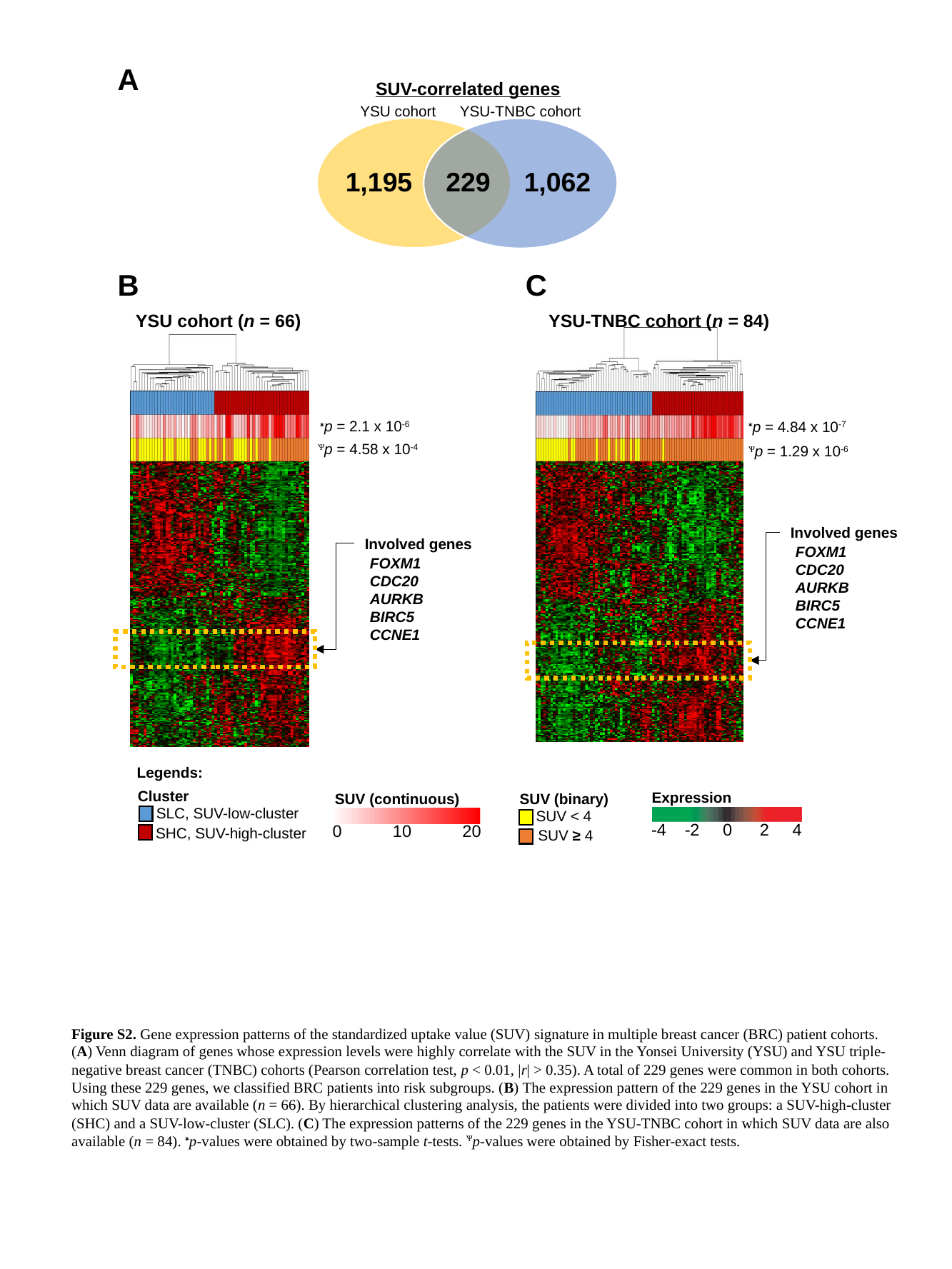

A
SUV-correlated genes
YSU cohort
YSU-TNBC cohort
1,195
229
1,062
B
C
YSU cohort (n = 66)
YSU-TNBC cohort (n = 84)
p = 2.1 x 10^-6^
p = 4.84 x 10^-7^
Ѱp = 4.58 x 10^-4^
Ѱp = 1.29 x 10^-6^
Involved genes
Involved genes
FOXM1
CDC20
AURKB
BIRC5
CCNE1
FOXM1
CDC20
AURKB
BIRC5
CCNE1
Legends:
Cluster
Expression
SUV (binary)
SUV (continuous)
SLC, SUV-low-cluster
SUV < 4
-4 -2 0 2 4
0 10 20
SHC, SUV-high-cluster
SUV ≥ 4
Figure S2. Gene expression patterns of the standardized uptake value (SUV) signature in multiple breast cancer (BRC) patient cohorts. (A) Venn diagram of genes whose expression levels were highly correlate with the SUV in the Yonsei University (YSU) and YSU triple-negative breast cancer (TNBC) cohorts (Pearson correlation test, p < 0.01, |r| > 0.35). A total of 229 genes were common in both cohorts. Using these 229 genes, we classified BRC patients into risk subgroups. (B) The expression pattern of the 229 genes in the YSU cohort in which SUV data are available (n = 66). By hierarchical clustering analysis, the patients were divided into two groups: a SUV-high-cluster (SHC) and a SUV-low-cluster (SLC). (C) The expression patterns of the 229 genes in the YSU-TNBC cohort in which SUV data are also available (n = 84). p-values were obtained by two-sample t-tests. Ѱp-values were obtained by Fisher-exact tests.

## Slide 3
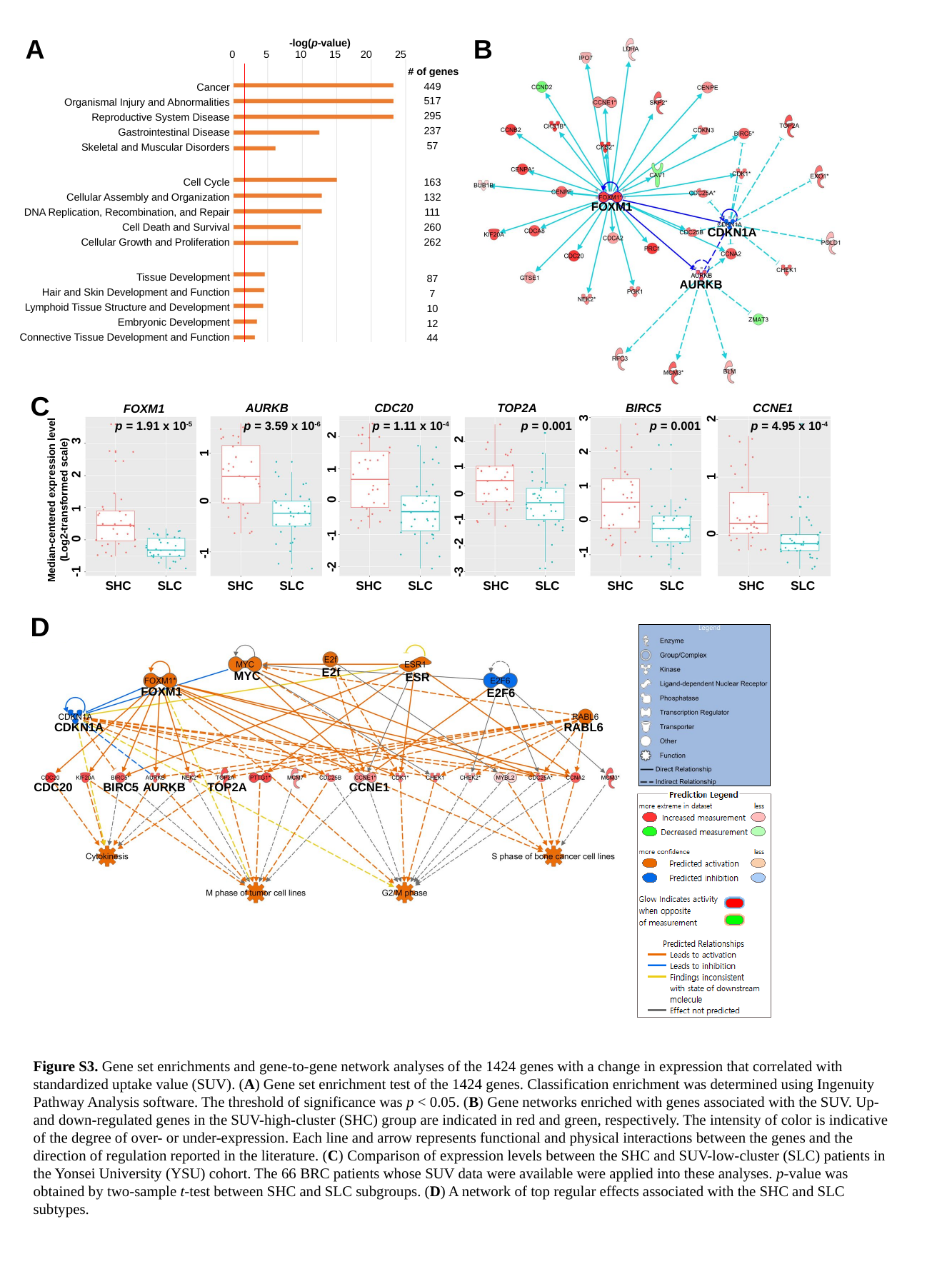

-log(p-value)
A
B
0 5 10 15 20 25
# of genes
449
517
295
237
57
Cancer
Organismal Injury and Abnormalities
Reproductive System Disease
Gastrointestinal Disease
Skeletal and Muscular Disorders
Cell Cycle
Cellular Assembly and Organization
DNA Replication, Recombination, and Repair
Cell Death and Survival
Cellular Growth and Proliferation
163
132
111
260
262
FOXM1
CDKN1A
Tissue Development
Hair and Skin Development and Function
Lymphoid Tissue Structure and Development
Embryonic Development
Connective Tissue Development and Function
87
7
10
12
44
AURKB
C
AURKB
CDC20
TOP2A
BIRC5
CCNE1
FOXM1
p = 1.91 x 10^-5^
p = 3.59 x 10^-6^
p = 1.11 x 10^-4^
p = 0.001
p = 0.001
p = 4.95 x 10^-4^
-1 0 1
 -1 0 1 2 3
 0 1 2
-2 -1 0 1 2
Median-centered expression level
(Log2-transformed scale)
-1 0 1 2 3
-3 -2 -1 0 1 2
SHC
SLC
SHC
SLC
SHC
SLC
SHC
SLC
SHC
SLC
SHC
SLC
D
E2f
MYC
ESR
FOXM1
E2F6
CDKN1A
RABL6
CDC20
BIRC5
AURKB
TOP2A
CCNE1
Figure S3. Gene set enrichments and gene-to-gene network analyses of the 1424 genes with a change in expression that correlated with standardized uptake value (SUV). (A) Gene set enrichment test of the 1424 genes. Classification enrichment was determined using Ingenuity Pathway Analysis software. The threshold of significance was p < 0.05. (B) Gene networks enriched with genes associated with the SUV. Up- and down-regulated genes in the SUV-high-cluster (SHC) group are indicated in red and green, respectively. The intensity of color is indicative of the degree of over- or under-expression. Each line and arrow represents functional and physical interactions between the genes and the direction of regulation reported in the literature. (C) Comparison of expression levels between the SHC and SUV-low-cluster (SLC) patients in the Yonsei University (YSU) cohort. The 66 BRC patients whose SUV data were available were applied into these analyses. p-value was obtained by two-sample t-test between SHC and SLC subgroups. (D) A network of top regular effects associated with the SHC and SLC subtypes.

## Slide 4
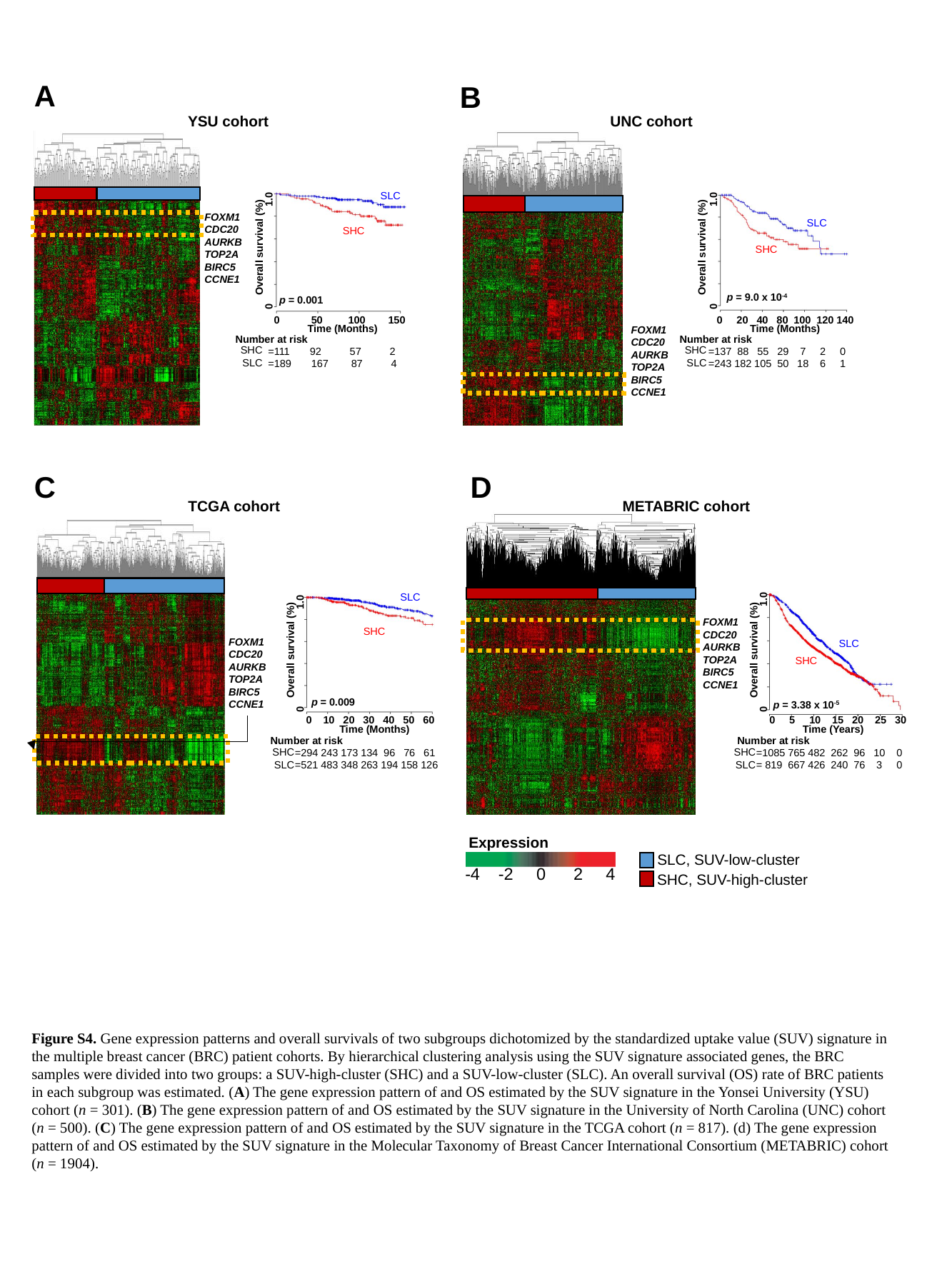

A
B
YSU cohort
UNC cohort
SLC
FOXM1
CDC20
AURKB
TOP2A
BIRC5
CCNE1
SLC
SHC
0 1.0
0 1.0
Overall survival (%)
Overall survival (%)
SHC
p = 9.0 x 10^-4^
p = 0.001
0 50 100 150
0 20 40 80 100 120 140
Time (Months)
Time (Months)
FOXM1
CDC20
AURKB
TOP2A
BIRC5
CCNE1
Number at risk
Number at risk
SHCSLC
SHCSLC
=111 92 57 2
=189 167 87 4
=137 88 55 29 7 2 0
=243 182 105 50 18 6 1
C
D
TCGA cohort
METABRIC cohort
SLC
FOXM1
CDC20
AURKB
TOP2A
BIRC5
CCNE1
SHC
FOXM1
CDC20
AURKB
TOP2A
BIRC5
CCNE1
SLC
0 1.0
0 1.0
Overall survival (%)
Overall survival (%)
SHC
p = 0.009
p = 3.38 x 10^-5^
0 10 20 30 40 50 60
0 5 10 15 20 25 30
Time (Months)
Time (Years)
Number at risk
Number at risk
SHCSLC
SHCSLC
=294 243 173 134 96 76 61
=521 483 348 263 194 158 126
=1085 765 482 262 96 10 0
= 819 667 426 240 76 3 0
Expression
SLC, SUV-low-cluster
-4 -2 0 2 4
SHC, SUV-high-cluster
Figure S4. Gene expression patterns and overall survivals of two subgroups dichotomized by the standardized uptake value (SUV) signature in the multiple breast cancer (BRC) patient cohorts. By hierarchical clustering analysis using the SUV signature associated genes, the BRC samples were divided into two groups: a SUV-high-cluster (SHC) and a SUV-low-cluster (SLC). An overall survival (OS) rate of BRC patients in each subgroup was estimated. (A) The gene expression pattern of and OS estimated by the SUV signature in the Yonsei University (YSU) cohort (n = 301). (B) The gene expression pattern of and OS estimated by the SUV signature in the University of North Carolina (UNC) cohort (n = 500). (C) The gene expression pattern of and OS estimated by the SUV signature in the TCGA cohort (n = 817). (d) The gene expression pattern of and OS estimated by the SUV signature in the Molecular Taxonomy of Breast Cancer International Consortium (METABRIC) cohort (n = 1904).

## Slide 5
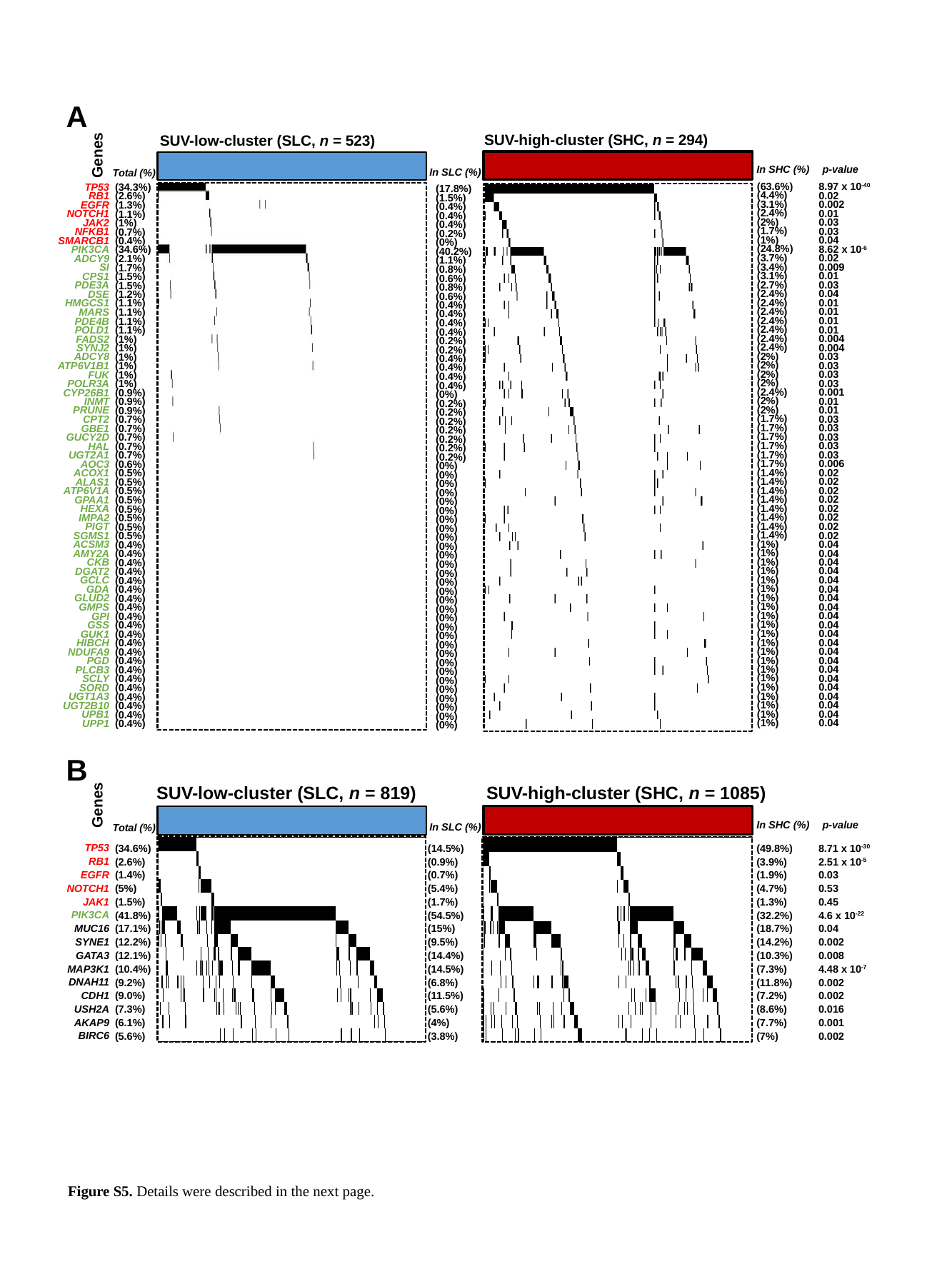

A
SUV-high-cluster (SHC, n = 294)
SUV-low-cluster (SLC, n = 523)
Genes
In SHC (%)
p-value
In SLC (%)
Total (%)
(63.6%)
(4.4%)
(3.1%)
(2.4%)
(2%)
(1.7%)
(1%)
(24.8%)
(3.7%)
(3.4%)
(3.1%)
(2.7%)
(2.4%)
(2.4%)
(2.4%)
(2.4%)
(2.4%)
(2.4%)
(2.4%)
(2%)
(2%)
(2%)
(2%)
(2.4%)
(2%)
(2%)
(1.7%)
(1.7%)
(1.7%)
(1.7%)
(1.7%)
(1.7%)
(1.4%)
(1.4%)
(1.4%)
(1.4%)
(1.4%)
(1.4%)
(1.4%)
(1.4%)
(1%)
(1%)
(1%)
(1%)
(1%)
(1%)
(1%)
(1%)
(1%)
(1%)
(1%)
(1%)
(1%)
(1%)
(1%)
(1%)
(1%)
(1%)
(1%)
(1%)
(1%)
8.97 x 10^-40^
0.02
0.002
0.01
0.03
0.03
0.04
8.62 x 10^-6^
0.02
0.009
0.01
0.03
0.04
0.01
0.01
0.01
0.01
0.004
0.004
0.03
0.03
0.03
0.03
0.001
0.01
0.01
0.03
0.03
0.03
0.03
0.03
0.006
0.02
0.02
0.02
0.02
0.02
0.02
0.02
0.02
0.04
0.04
0.04
0.04
0.04
0.04
0.04
0.04
0.04
0.04
0.04
0.04
0.04
0.04
0.04
0.04
0.04
0.04
0.04
0.04
0.04
TP53
RB1
EGFR
NOTCH1
JAK2
NFKB1
SMARCB1
PIK3CA
ADCY9
SI
CPS1
PDE3A
DSE
HMGCS1
MARS
PDE4B
POLD1
FADS2
SYNJ2
ADCY8
ATP6V1B1
FUK
POLR3A
CYP26B1
INMT
PRUNE
CPT2
GBE1
GUCY2D
HAL
UGT2A1
AOC3
ACOX1
ALAS1
ATP6V1A
GPAA1
HEXA
IMPA2
PIGT
SGMS1
ACSM3
AMY2A
CKB
DGAT2
GCLC
GDA
GLUD2
GMPS
GPI
GSS
GUK1
HIBCH
NDUFA9
PGD
PLCB3
SCLY
SORD
UGT1A3
UGT2B10
UPB1
UPP1
 (34.3%)
 (2.6%)
 (1.3%)
 (1.1%)
 (1%)
 (0.7%)
 (0.4%)
 (34.6%)
 (2.1%)
 (1.7%)
 (1.5%)
 (1.5%)
 (1.2%)
 (1.1%)
 (1.1%)
 (1.1%)
 (1.1%)
 (1%)
 (1%)
 (1%)
 (1%)
 (1%)
 (1%)
 (0.9%)
 (0.9%)
 (0.9%)
 (0.7%)
 (0.7%)
 (0.7%)
 (0.7%)
 (0.7%)
 (0.6%)
 (0.5%)
 (0.5%)
 (0.5%)
 (0.5%)
 (0.5%)
 (0.5%)
 (0.5%)
 (0.5%)
 (0.4%)
 (0.4%)
 (0.4%)
 (0.4%)
 (0.4%)
 (0.4%)
 (0.4%)
 (0.4%)
 (0.4%)
 (0.4%)
 (0.4%)
 (0.4%)
 (0.4%)
 (0.4%)
 (0.4%)
 (0.4%)
 (0.4%)
 (0.4%)
 (0.4%)
 (0.4%)
 (0.4%)
(17.8%)
(1.5%)
(0.4%)
(0.4%)
(0.4%)
(0.2%)
(0%)
(40.2%)
(1.1%)
(0.8%)
(0.6%)
(0.8%)
(0.6%)
(0.4%)
(0.4%)
(0.4%)
(0.4%)
(0.2%)
(0.2%)
(0.4%)
(0.4%)
(0.4%)
(0.4%)
(0%)
(0.2%)
(0.2%)
(0.2%)
(0.2%)
(0.2%)
(0.2%)
(0.2%)
(0%)
(0%)
(0%)
(0%)
(0%)
(0%)
(0%)
(0%)
(0%)
(0%)
(0%)
(0%)
(0%)
(0%)
(0%)
(0%)
(0%)
(0%)
(0%)
(0%)
(0%)
(0%)
(0%)
(0%)
(0%)
(0%)
(0%)
(0%)
(0%)
(0%)
B
SUV-low-cluster (SLC, n = 819)
SUV-high-cluster (SHC, n = 1085)
Genes
In SHC (%)
p-value
In SLC (%)
Total (%)
TP53
RB1
EGFR
NOTCH1
JAK1
PIK3CA
MUC16
SYNE1
GATA3
MAP3K1
DNAH11
CDH1
USH2A
AKAP9
BIRC6
 (34.6%)
 (2.6%)
 (1.4%)
 (5%)
 (1.5%)
 (41.8%)
 (17.1%)
 (12.2%)
 (12.1%)
 (10.4%)
 (9.2%)
 (9.0%)
 (7.3%)
 (6.1%)
 (5.6%)
 (14.5%)
 (0.9%)
 (0.7%)
 (5.4%)
 (1.7%)
 (54.5%)
 (15%)
 (9.5%)
 (14.4%)
 (14.5%)
 (6.8%)
 (11.5%)
 (5.6%)
 (4%)
 (3.8%)
 (49.8%)
 (3.9%)
 (1.9%)
 (4.7%)
 (1.3%)
 (32.2%)
 (18.7%)
 (14.2%)
 (10.3%)
 (7.3%)
 (11.8%)
 (7.2%)
 (8.6%)
 (7.7%)
 (7%)
8.71 x 10^-30^
2.51 x 10^-5^
0.03
0.53
0.45
4.6 x 10^-22^
0.04
0.002
0.008
4.48 x 10^-7^
0.002
0.002
0.016
0.001
0.002
Figure S5. Details were described in the next page.

## Slide 6
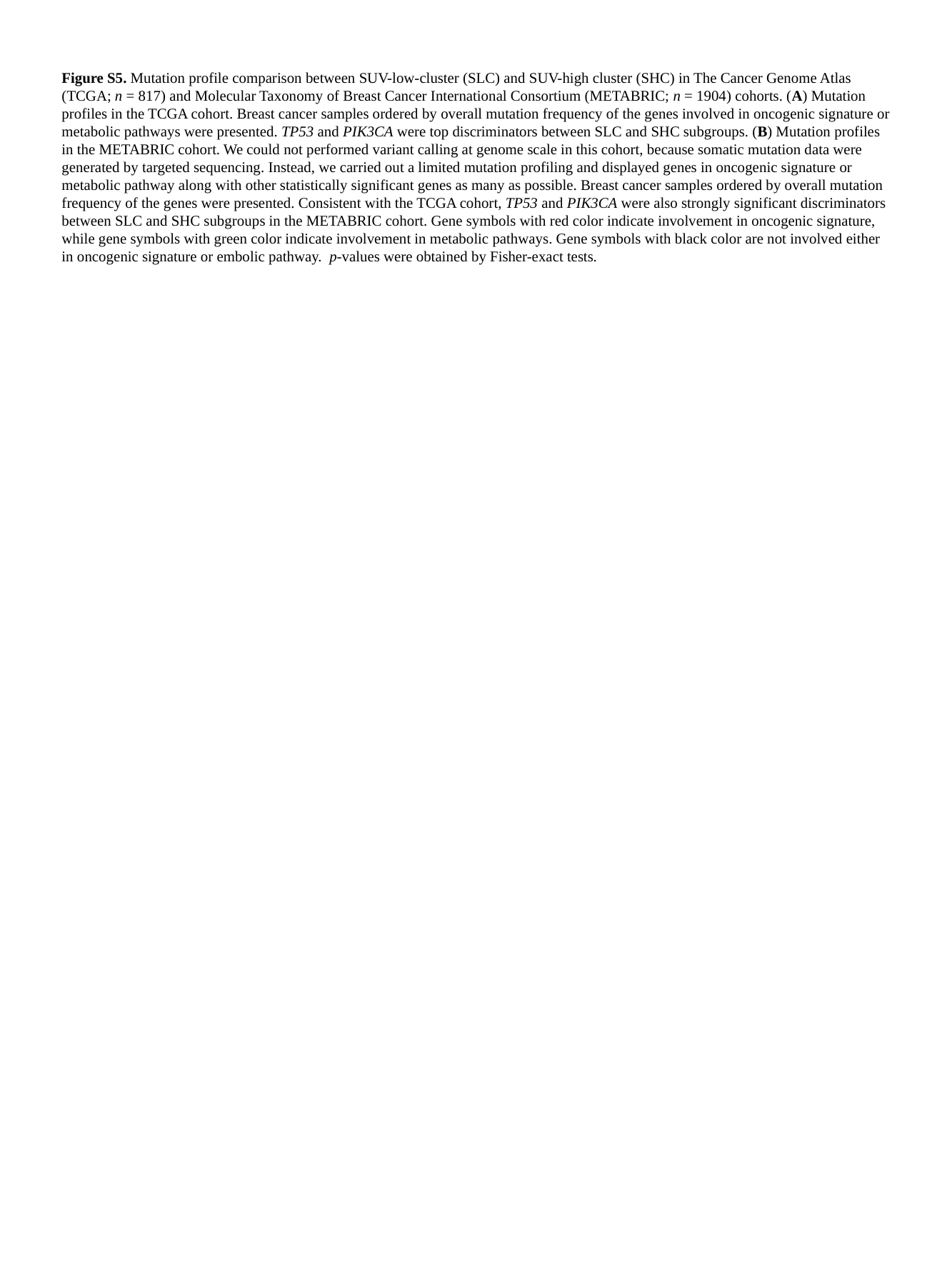

Figure S5. Mutation profile comparison between SUV-low-cluster (SLC) and SUV-high cluster (SHC) in The Cancer Genome Atlas (TCGA; n = 817) and Molecular Taxonomy of Breast Cancer International Consortium (METABRIC; n = 1904) cohorts. (A) Mutation profiles in the TCGA cohort. Breast cancer samples ordered by overall mutation frequency of the genes involved in oncogenic signature or metabolic pathways were presented. TP53 and PIK3CA were top discriminators between SLC and SHC subgroups. (B) Mutation profiles in the METABRIC cohort. We could not performed variant calling at genome scale in this cohort, because somatic mutation data were generated by targeted sequencing. Instead, we carried out a limited mutation profiling and displayed genes in oncogenic signature or metabolic pathway along with other statistically significant genes as many as possible. Breast cancer samples ordered by overall mutation frequency of the genes were presented. Consistent with the TCGA cohort, TP53 and PIK3CA were also strongly significant discriminators between SLC and SHC subgroups in the METABRIC cohort. Gene symbols with red color indicate involvement in oncogenic signature, while gene symbols with green color indicate involvement in metabolic pathways. Gene symbols with black color are not involved either in oncogenic signature or embolic pathway. p-values were obtained by Fisher-exact tests.

## Slide 7
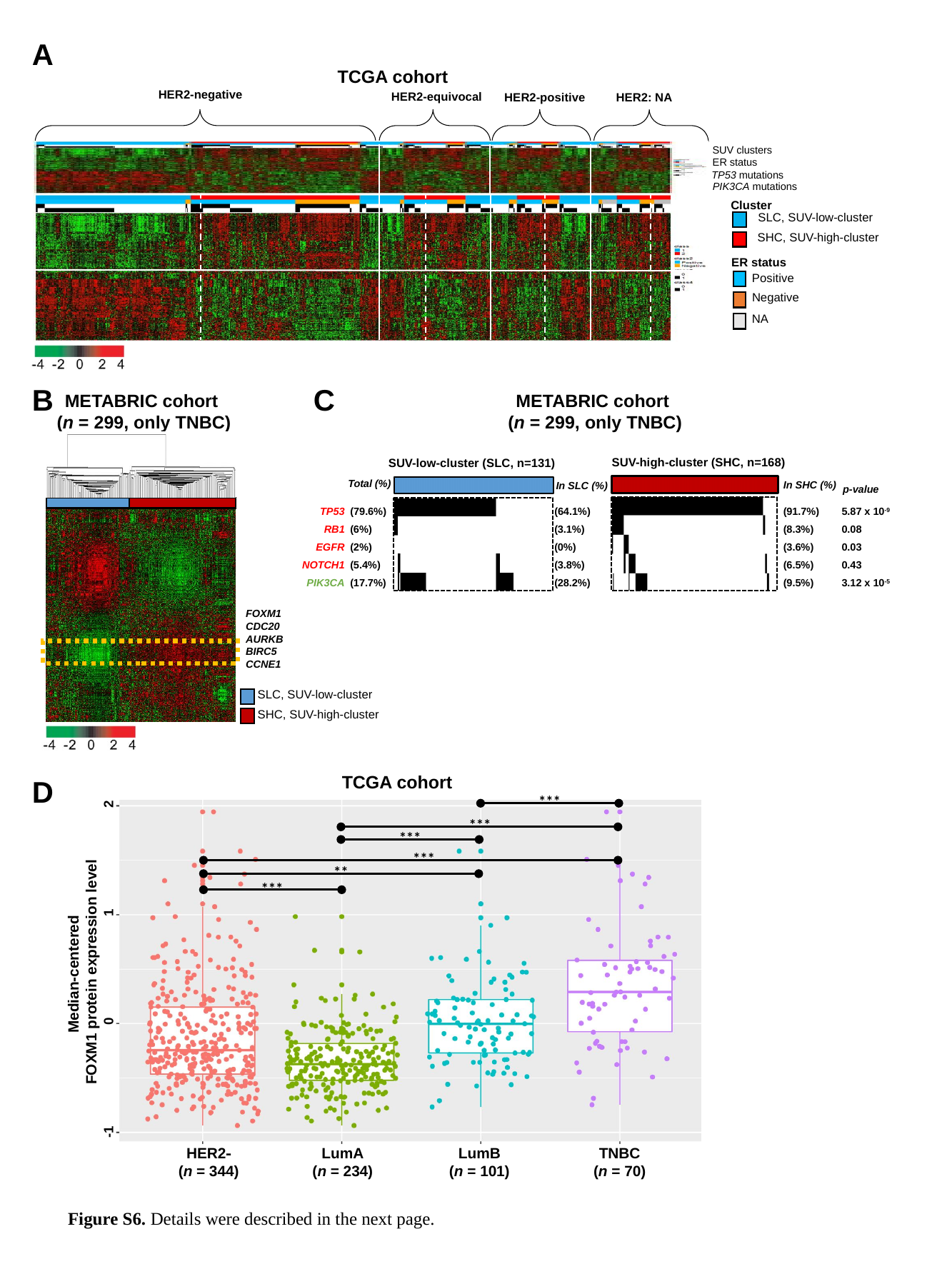

A
TCGA cohort
HER2-negative
HER2-equivocal
HER2-positive
HER2: NA
SUV clusters
ER status
TP53 mutations
PIK3CA mutations
Cluster
SLC, SUV-low-cluster
SHC, SUV-high-cluster
ER status
Positive
Negative
NA
B
C
METABRIC cohort
(n = 299, only TNBC)
METABRIC cohort
(n = 299, only TNBC)
SUV-high-cluster (SHC, n=168)
SUV-low-cluster (SLC, n=131)
Total (%)
In SHC (%)
In SLC (%)
p-value
TP53
RB1
EGFR
NOTCH1
PIK3CA
5.87 x 10^-9^
0.08
0.03
0.43
3.12 x 10^-5^
 (79.6%)
 (6%)
 (2%)
 (5.4%)
 (17.7%)
 (64.1%)
 (3.1%)
 (0%)
 (3.8%)
 (28.2%)
 (91.7%)
 (8.3%)
 (3.6%)
 (6.5%)
 (9.5%)
FOXM1
CDC20
AURKB
BIRC5
CCNE1
SLC, SUV-low-cluster
SHC, SUV-high-cluster
TCGA cohort
D
***
***
***
***
**
***
Median-centered
FOXM1 protein expression level
-1 0 1 2
HER2−
(n = 344)
LumA
(n = 234)
LumB
(n = 101)
TNBC
(n = 70)
Figure S6. Details were described in the next page.

## Slide 8
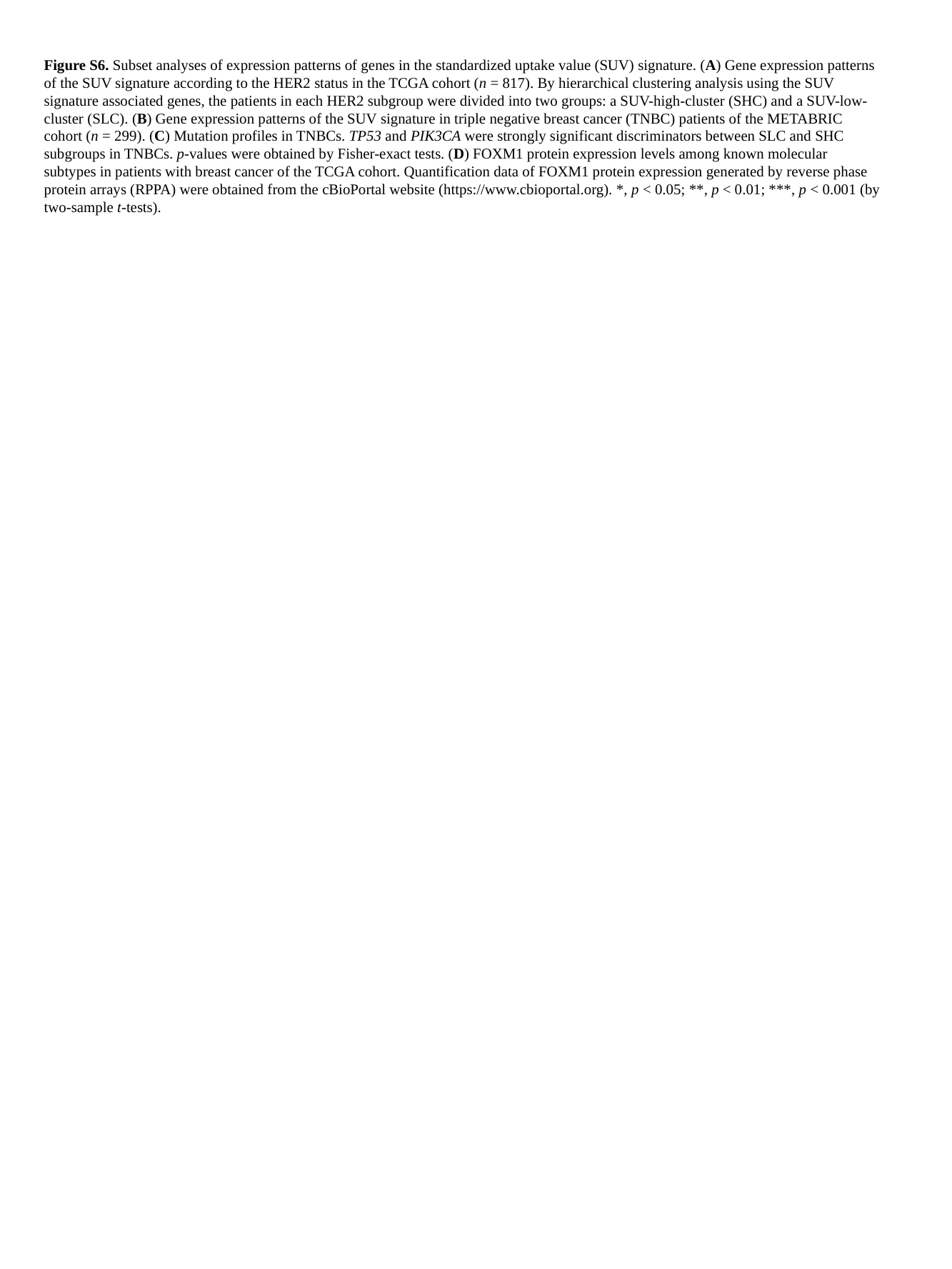

Figure S6. Subset analyses of expression patterns of genes in the standardized uptake value (SUV) signature. (A) Gene expression patterns of the SUV signature according to the HER2 status in the TCGA cohort (n = 817). By hierarchical clustering analysis using the SUV signature associated genes, the patients in each HER2 subgroup were divided into two groups: a SUV-high-cluster (SHC) and a SUV-low-cluster (SLC). (B) Gene expression patterns of the SUV signature in triple negative breast cancer (TNBC) patients of the METABRIC cohort (n = 299). (C) Mutation profiles in TNBCs. TP53 and PIK3CA were strongly significant discriminators between SLC and SHC subgroups in TNBCs. p-values were obtained by Fisher-exact tests. (D) FOXM1 protein expression levels among known molecular subtypes in patients with breast cancer of the TCGA cohort. Quantification data of FOXM1 protein expression generated by reverse phase protein arrays (RPPA) were obtained from the cBioPortal website (https://www.cbioportal.org). *, p < 0.05; **, p < 0.01; ***, p < 0.001 (by two-sample t-tests).

## Slide 9
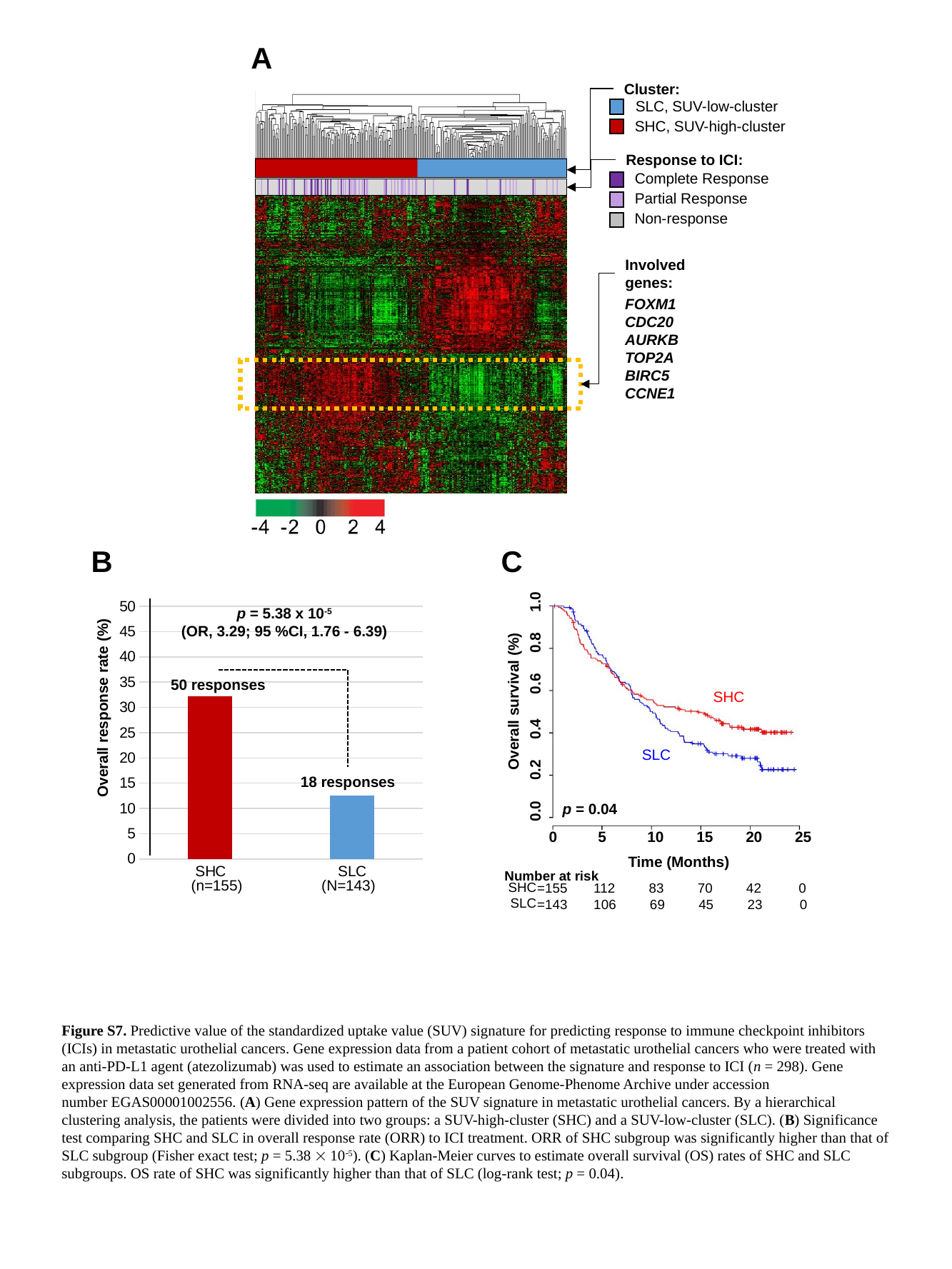

A
Cluster:
SLC, SUV-low-cluster
SHC, SUV-high-cluster
Response to ICI:
Complete Response
Partial Response
Non-response
Involved genes:
FOXM1
CDC20
AURKB
TOP2A
BIRC5
CCNE1
B
C
### Chart
| Category | %ratio |
|---|---|
| SHC | 32.25806451612903 |
| SLC | 12.587412587412588 |p = 5.38 x 10^-5^
(OR, 3.29; 95 %CI, 1.76 - 6.39)
50 responses
SHC
Overall survival (%)
0.0 0.2 0.4 0.6 0.8 1.0
Overall response rate (%)
SLC
18 responses
p = 0.04
0 5 10 15 20 25
Time (Months)
Number at risk
(n=155)
(N=143)
SHC
SLC
=155 112 83 70 42 0
=143 106 69 45 23 0
Figure S7. Predictive value of the standardized uptake value (SUV) signature for predicting response to immune checkpoint inhibitors (ICIs) in metastatic urothelial cancers. Gene expression data from a patient cohort of metastatic urothelial cancers who were treated with an anti-PD-L1 agent (atezolizumab) was used to estimate an association between the signature and response to ICI (n = 298). Gene expression data set generated from RNA-seq are available at the European Genome-Phenome Archive under accession number EGAS00001002556. (A) Gene expression pattern of the SUV signature in metastatic urothelial cancers. By a hierarchical clustering analysis, the patients were divided into two groups: a SUV-high-cluster (SHC) and a SUV-low-cluster (SLC). (B) Significance test comparing SHC and SLC in overall response rate (ORR) to ICI treatment. ORR of SHC subgroup was significantly higher than that of SLC subgroup (Fisher exact test; p = 5.38 × 10^−5^). (C) Kaplan-Meier curves to estimate overall survival (OS) rates of SHC and SLC subgroups. OS rate of SHC was significantly higher than that of SLC (log-rank test; p = 0.04).

## Slide 10
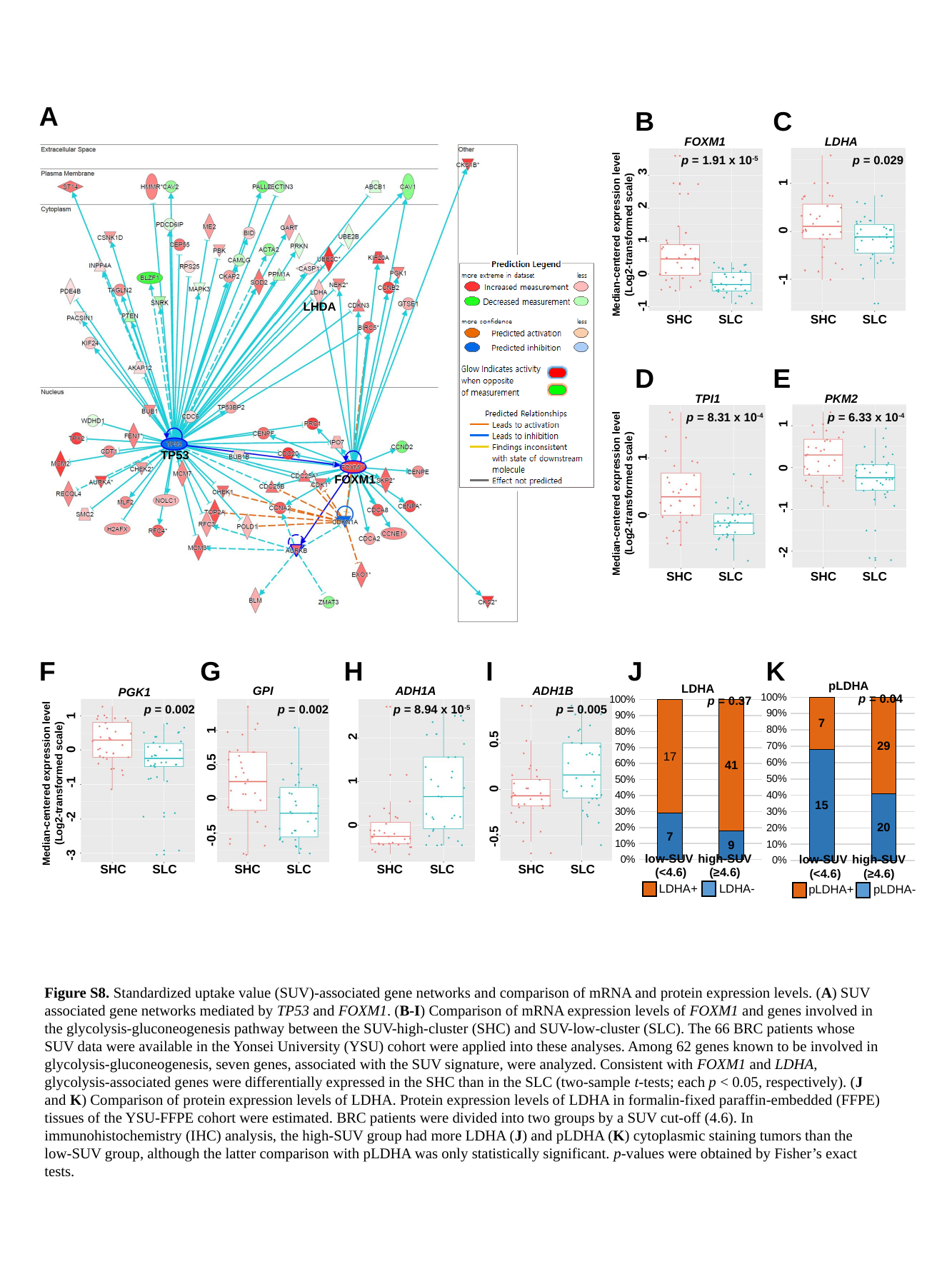

A
B
C
FOXM1
LDHA
p = 1.91 x 10^-5^
p = 0.029
 -1 0 1
Median-centered expression level
(Log2-transformed scale)
-1 0 1 2 3
LHDA
SHC
SLC
SHC
SLC
D
E
TPI1
PKM2
p = 8.31 x 10^-4^
p = 6.33 x 10^-4^
TP53
 0 1
-2 -1 0 1
FOXM1
Median-centered expression level
(Log2-transformed scale)
SHC
SLC
SHC
SLC
F
G
H
I
J
K
pLDHA
LDHA
GPI
ADH1A
ADH1B
PGK1
p = 0.04
p = 0.37
### Chart
| Category | pLDHA-neg | pLDHA-pos |
|---|---|---|
| low SUV (<4.6) | 15.0 | 7.0 |
| high SUV (>=4.6) | 20.0 | 29.0 |
### Chart
| Category | LDHA-neg | LDHA-pos |
|---|---|---|
| low SUV (<4.6) | 7.0 | 17.0 |
| high SUV (>=4.6) | 9.0 | 41.0 |p = 0.002
p = 0.002
p = 8.94 x 10^-5^
p = 0.005
-0.5 0 0.5 1
 0 1 2
Median-centered expression level
(Log2-transformed scale)
-3 -2 -1 0 1
 -0.5 0 0.5
low-SUV
(<4.6)
high-SUV
(≥4.6)
low-SUV
(<4.6)
high-SUV
(≥4.6)
SHC
SLC
SHC
SLC
SHC
SLC
SHC
SLC
LDHA-
LDHA+
pLDHA-
pLDHA+
Figure S8. Standardized uptake value (SUV)-associated gene networks and comparison of mRNA and protein expression levels. (A) SUV associated gene networks mediated by TP53 and FOXM1. (B-I) Comparison of mRNA expression levels of FOXM1 and genes involved in the glycolysis-gluconeogenesis pathway between the SUV-high-cluster (SHC) and SUV-low-cluster (SLC). The 66 BRC patients whose SUV data were available in the Yonsei University (YSU) cohort were applied into these analyses. Among 62 genes known to be involved in glycolysis-gluconeogenesis, seven genes, associated with the SUV signature, were analyzed. Consistent with FOXM1 and LDHA, glycolysis-associated genes were differentially expressed in the SHC than in the SLC (two-sample t-tests; each p < 0.05, respectively). (J and K) Comparison of protein expression levels of LDHA. Protein expression levels of LDHA in formalin-fixed paraffin-embedded (FFPE) tissues of the YSU-FFPE cohort were estimated. BRC patients were divided into two groups by a SUV cut-off (4.6). In immunohistochemistry (IHC) analysis, the high-SUV group had more LDHA (J) and pLDHA (K) cytoplasmic staining tumors than the low-SUV group, although the latter comparison with pLDHA was only statistically significant. p-values were obtained by Fisher’s exact tests.

## Slide 11
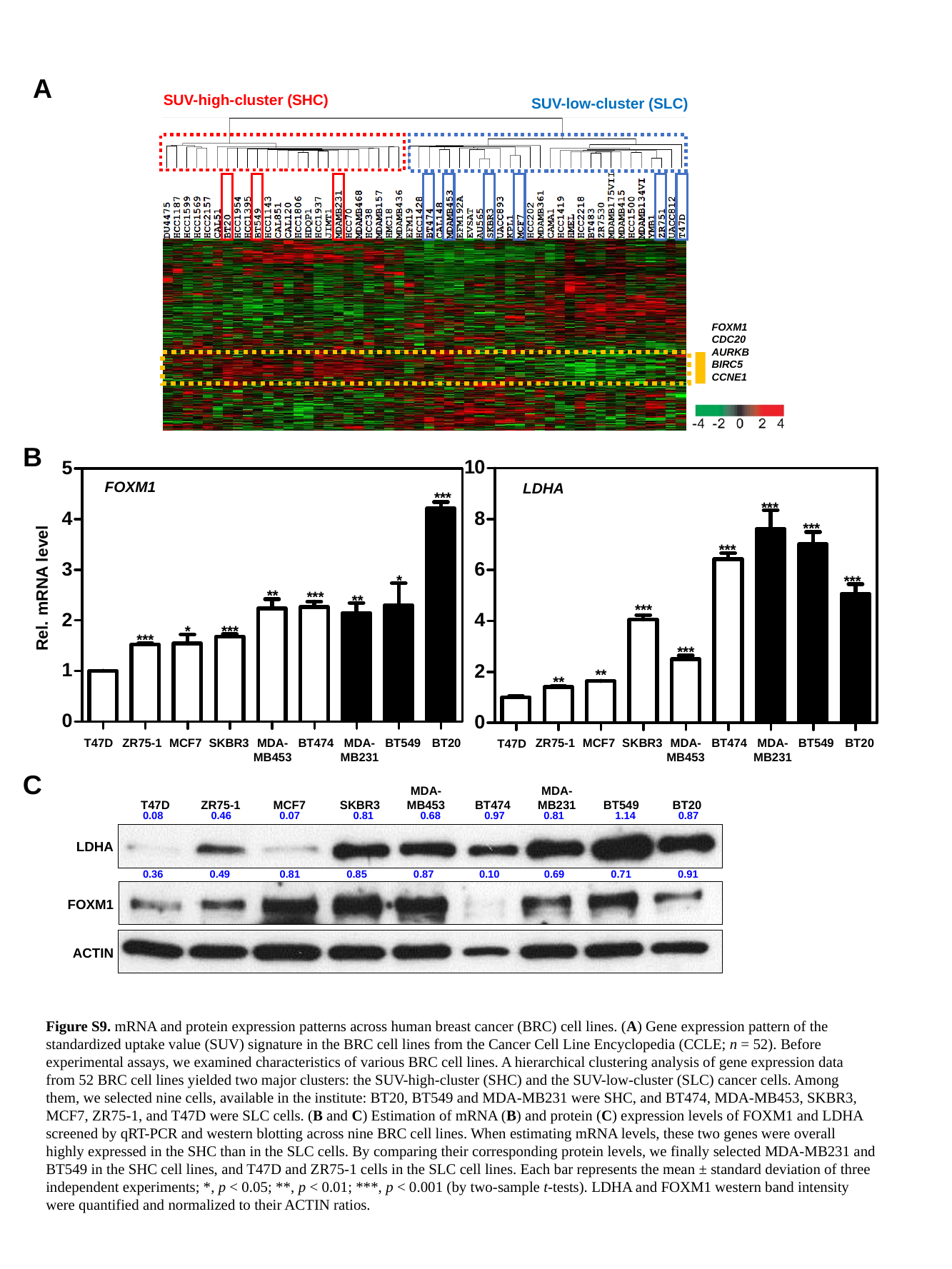

A
SUV-high-cluster (SHC)
SUV-low-cluster (SLC)
FOXM1
CDC20
AURKB
BIRC5
CCNE1
B
FOXM1
LDHA
***
***
***
***
*
***
Rel. mRNA level
**
***
**
***
***
*
***
***
**
**
ZR75-1
MCF7
SKBR3
BT474
BT549
BT20
MDA-
MB453
MDA-MB231
ZR75-1
MCF7
SKBR3
BT474
BT549
BT20
MDA-MB453
MDA-MB231
T47D
T47D
C
MDA-
MB453
MDA-
MB231
T47D
ZR75-1
MCF7
SKBR3
BT474
BT549
BT20
0.08
0.46
0.07
0.81
0.68
0.97
0.81
1.14
0.87
LDHA
0.36
0.49
0.81
0.85
0.87
0.10
0.69
0.71
0.91
FOXM1
ACTIN
Figure S9. mRNA and protein expression patterns across human breast cancer (BRC) cell lines. (A) Gene expression pattern of the standardized uptake value (SUV) signature in the BRC cell lines from the Cancer Cell Line Encyclopedia (CCLE; n = 52). Before experimental assays, we examined characteristics of various BRC cell lines. A hierarchical clustering analysis of gene expression data from 52 BRC cell lines yielded two major clusters: the SUV-high-cluster (SHC) and the SUV-low-cluster (SLC) cancer cells. Among them, we selected nine cells, available in the institute: BT20, BT549 and MDA-MB231 were SHC, and BT474, MDA-MB453, SKBR3, MCF7, ZR75-1, and T47D were SLC cells. (B and C) Estimation of mRNA (B) and protein (C) expression levels of FOXM1 and LDHA screened by qRT-PCR and western blotting across nine BRC cell lines. When estimating mRNA levels, these two genes were overall highly expressed in the SHC than in the SLC cells. By comparing their corresponding protein levels, we finally selected MDA-MB231 and BT549 in the SHC cell lines, and T47D and ZR75-1 cells in the SLC cell lines. Each bar represents the mean ± standard deviation of three independent experiments; *, p < 0.05; **, p < 0.01; ***, p < 0.001 (by two-sample t-tests). LDHA and FOXM1 western band intensity were quantified and normalized to their ACTIN ratios.

## Slide 12
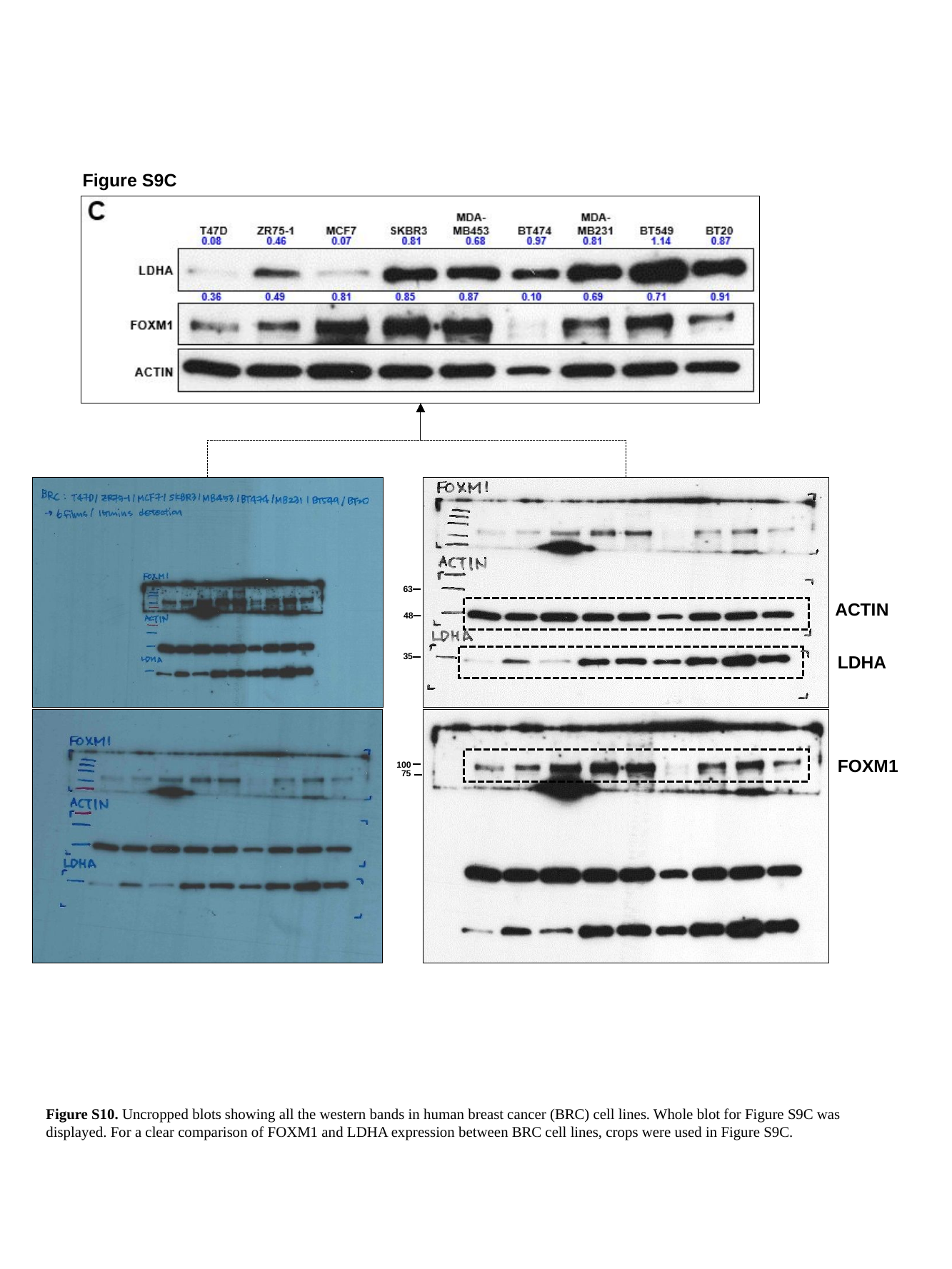

Figure S9C
63
ACTIN
48
35
LDHA
FOXM1
100
75
Figure S10. Uncropped blots showing all the western bands in human breast cancer (BRC) cell lines. Whole blot for Figure S9C was displayed. For a clear comparison of FOXM1 and LDHA expression between BRC cell lines, crops were used in Figure S9C.

## Slide 13
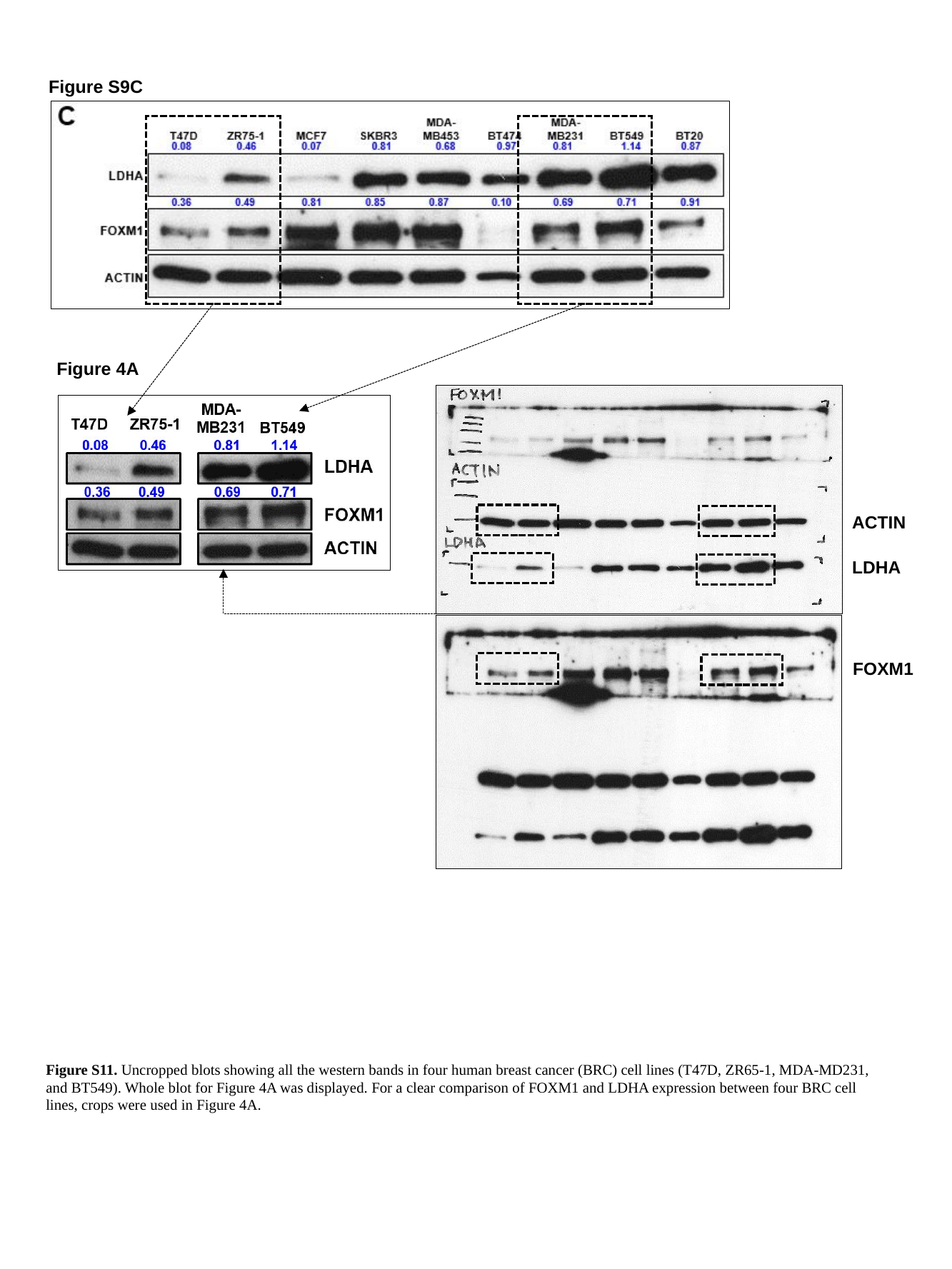

Figure S9C
Figure 4A
ACTIN
LDHA
FOXM1
Figure S11. Uncropped blots showing all the western bands in four human breast cancer (BRC) cell lines (T47D, ZR65-1, MDA-MD231, and BT549). Whole blot for Figure 4A was displayed. For a clear comparison of FOXM1 and LDHA expression between four BRC cell lines, crops were used in Figure 4A.

## Slide 14
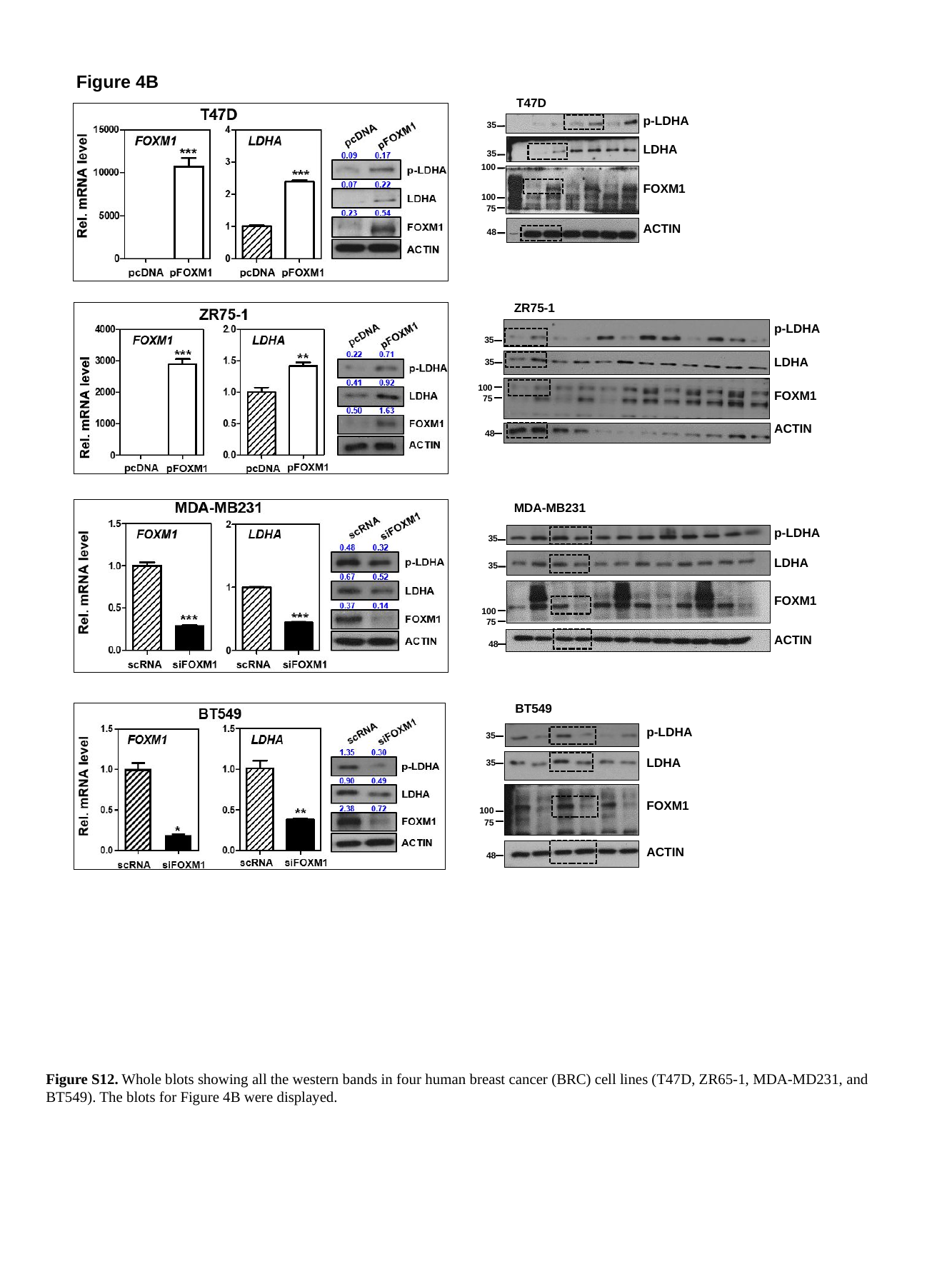

Figure 4B
T47D
p-LDHA
35
LDHA
35
100
FOXM1
100
75
ACTIN
48
ZR75-1
p-LDHA
35
LDHA
35
100
FOXM1
75
ACTIN
48
MDA-MB231
p-LDHA
35
LDHA
35
FOXM1
100
75
ACTIN
48
BT549
p-LDHA
35
LDHA
35
FOXM1
100
75
ACTIN
48
Figure S12. Whole blots showing all the western bands in four human breast cancer (BRC) cell lines (T47D, ZR65-1, MDA-MD231, and BT549). The blots for Figure 4B were displayed.
