# Supplementary material for: Genomic Signature of the Standardized Uptake Value in 18F-Fluorodeoxyglucose Positron Emission Tomography in Breast Cancer
Source: Cancers (Basel). 2020 Feb 20;12(2):497. doi: 10.3390/cancers12020497 (PMC7072341; doi:10.3390/cancers12020497)
Supplement: Supplementary file 1 [file cancers-12-00497-s001.zip › Supplementary_tables.doc]

**Supplementary Tables**

**Table S1.** 229 genes significantly correlated with the SUV.

| **Genes** | **Entrez Gene ID** | **Type(s)** | **YSU cohort (n=66)** | | **YSU-TNBC cohort (n=88)** | |
| --- | --- | --- | --- | --- | --- | --- |
|  |  |  | ***r*-value** | ***P*-value** | ***r*-value** | ***P*-value** |
| *ABCA6* | 23460 | transporter | -0.51 | 1.00E-05 | -0.51 | 1.00E-05 |
| *ABI3BP* | 25890 | other | -0.53 | 1.00E-05 | -0.52 | 1.00E-05 |
| *ABLIM3* | 22885 | other | -0.4 | 9.10E-04 | -0.42 | 6.00E-04 |
| *ACTA2* | 59 | other | -0.46 | 1.30E-04 | -0.49 | 2.00E-05 |
| *ADH1B* | 125 | enzyme | -0.39 | 1.37E-03 | -0.46 | 1.10E-04 |
| *AHNAK* | 79026 | other | -0.48 | 4.00E-05 | -0.36 | 4.58E-03 |
| *ANK2* | 287 | other | -0.36 | 2.64E-03 | -0.57 | 4.06E-07 |
| *ANKRD30A* | 91074 | other | -0.37 | 2.26E-03 | -0.35 | 4.91E-03 |
| *APOD* | 347 | transporter | -0.42 | 4.40E-04 | -0.46 | 9.00E-05 |
| *ARHGAP24* | 83478 | other | -0.35 | 4.01E-03 | -0.39 | 1.49E-03 |
| *ASF1B* | 55723 | other | 0.47 | 8.00E-05 | 0.39 | 1.72E-03 |
| *ATG4D* | 84971 | peptidase | 0.41 | 6.40E-04 | 0.41 | 8.20E-04 |
| *AURKB* | 9212 | kinase | 0.39 | 1.42E-03 | 0.4 | 1.31E-03 |
| *BCR* | 613 | kinase | 0.37 | 2.07E-03 | 0.49 | 3.00E-05 |
| *BIRC5* | 332 | other | 0.42 | 1.49E-03 | 0.6 | 4.70E-09 |
| *BLM* | 641 | enzyme | 0.41 | 6.30E-04 | 0.46 | 1.20E-04 |
| *BUB1* | 699 | kinase | 0.46 | 1.00E-04 | 0.41 | 7.20E-04 |
| *BUB1B* | 701 | kinase | 0.4 | 8.20E-04 | 0.44 | 2.90E-04 |
| *C14orf28* | 122525 | other | -0.46 | 1.20E-04 | -0.43 | 4.20E-04 |
| *C15orf42* | 90381 | other | 0.37 | 2.06E-03 | 0.41 | 6.80E-04 |
| *C17orf91* | 84981 | other | -0.42 | 4.40E-04 | -0.38 | 2.24E-03 |
| *C19orf28* | 100128569 | other | 0.36 | 3.12E-03 | 0.42 | 6.20E-04 |
| *C21orf34* | 388815 | other | -0.39 | 1.24E-03 | -0.47 | 7.00E-05 |
| *C3orf26* | 84319 | other | 0.37 | 2.64E-03 | 0.43 | 3.70E-04 |
| *C4orf34* | 201895 | other | -0.44 | 2.20E-04 | -0.45 | 1.60E-04 |
| *CA9* | 768 | enzyme | 0.49 | 3.00E-05 | 0.41 | 7.00E-04 |
| *CARD9* | 64170 | other | 0.42 | 4.70E-04 | 0.37 | 3.09E-03 |
| *CAV1* | 857 | transmembrane receptor | -0.45 | 1.50E-04 | -0.45 | 1.70E-04 |
| *CBX2* | 84733 | transcription regulator | 0.38 | 1.88E-03 | 0.6 | 2.69E-09 |
| *CCDC46* | 201134 | other | -0.36 | 3.46E-03 | -0.41 | 8.60E-04 |
| *CCNE1* | 898 | transcription regulator | 0.41 | 9.63E-04 | 0.36 | 4.72E-03 |
| *CCT3* | 7203 | other | 0.5 | 2.00E-05 | 0.36 | 4.04E-03 |
| *CD34* | 947 | other | -0.37 | 2.25E-03 | -0.43 | 3.40E-04 |
| *CD93* | 22918 | other | -0.36 | 2.98E-03 | -0.38 | 2.44E-03 |
| *CDC20* | 991 | other | 0.41 | 6.20E-04 | 0.48 | 4.00E-05 |
| *CDC25A* | 993 | phosphatase | 0.51 | 5.00E-05 | 0.53 | 4.91E-06 |
| *CDCA2* | 157313 | other | 0.47 | 7.00E-05 | 0.39 | 1.59E-03 |
| *CDCA3* | 83461 | other | 0.46 | 1.20E-04 | 0.51 | 1.00E-05 |
| *CDCA5* | 113130 | other | 0.52 | 1.00E-05 | 0.49 | 3.00E-05 |
| *CDH5* | 1003 | other | -0.45 | 1.60E-04 | -0.37 | 3.00E-03 |
| *CDT1* | 81620 | other | 0.36 | 2.67E-03 | 0.39 | 1.50E-03 |
| *CENPA* | 1058 | other | 0.47 | 8.00E-05 | 0.5 | 2.00E-05 |
| *CENPF* | 1063 | other | 0.44 | 2.10E-04 | 0.42 | 6.30E-04 |
| *CENPO* | 79172 | other | 0.45 | 1.80E-04 | 0.41 | 8.10E-04 |
| *CHAF1B* | 8208 | other | 0.36 | 3.04E-03 | 0.44 | 2.90E-04 |
| *CHCHD1* | 118487 | other | 0.46 | 1.10E-04 | 0.42 | 5.90E-04 |
| *CHD9* | 80205 | other | -0.39 | 1.08E-03 | -0.38 | 2.07E-03 |
| *CHEK2* | 11200 | kinase | 0.41 | 7.85E-04 | 0.42 | 4.70E-04 |
| *CILP* | 8483 | phosphatase | -0.45 | 2.30E-04 | -0.54 | 2.95E-06 |
| *CKAP2L* | 150468 | other | 0.42 | 4.10E-04 | 0.47 | 6.00E-05 |
| *CKAP5* | 9793 | transcription regulator | 0.37 | 2.28E-03 | 0.35 | 5.51E-03 |
| *COL14A1* | 7373 | other | -0.42 | 4.00E-04 | -0.53 | 4.51E-06 |
| *COL6A3* | 1293 | other | -0.4 | 1.40E-03 | -0.4 | 1.10E-03 |
| *CPE* | 1363 | peptidase | -0.38 | 1.76E-03 | -0.48 | 4.00E-05 |
| *CSNK1D* | 1453 | kinase | 0.43 | 3.20E-04 | 0.35 | 4.95E-03 |
| *CSTB* | 1476 | peptidase | 0.42 | 3.90E-04 | 0.36 | 4.42E-03 |
| *CXCL12* | 6387 | cytokine | -0.38 | 1.91E-03 | -0.36 | 4.58E-03 |
| *DAAM1* | 23002 | other | -0.41 | 6.30E-04 | -0.47 | 8.00E-05 |
| *DCN* | 1634 | other | -0.36 | 3.33E-03 | -0.51 | 1.00E-05 |
| *DDX11* | 1663 | enzyme | 0.36 | 2.65E-03 | 0.44 | 2.40E-04 |
| *DKC1* | 1736 | enzyme | 0.45 | 1.50E-04 | 0.37 | 3.46E-03 |
| *DLC1* | 10395 | other | -0.36 | 2.69E-03 | -0.52 | 1.00E-05 |
| *DOT1L* | 84444 | phosphatase | 0.37 | 2.21E-03 | 0.35 | 5.33E-03 |
| *DPT* | 1805 | other | -0.37 | 2.48E-03 | -0.42 | 5.80E-04 |
| *DPYSL3* | 1809 | enzyme | -0.38 | 1.52E-03 | -0.41 | 8.90E-04 |
| *DST* | 667 | other | -0.36 | 2.81E-03 | -0.38 | 2.59E-03 |
| *DUS1L* | 64118 | other | 0.43 | 3.50E-04 | 0.41 | 6.90E-04 |
| *EBF1* | 1879 | transcription regulator | -0.59 | 2.43E-07 | -0.58 | 1.83E-07 |
| *ECE2* | 9718 | peptidase | 0.42 | 5.00E-04 | 0.39 | 1.77E-03 |
| *ECM2* | 1842 | other | -0.39 | 1.18E-03 | -0.55 | 1.06E-06 |
| *EDNRA* | 1909 | transmembrane receptor | -0.36 | 3.26E-03 | -0.48 | 4.00E-05 |
| *ESRRA* | 2101 | ligand-dependent nuclear receptor | 0.45 | 1.30E-04 | 0.36 | 4.44E-03 |
| *F2RL2* | 2151 | G-protein coupled receptor | -0.4 | 7.90E-04 | -0.45 | 1.50E-04 |
| *FAM64A* | 54478 | other | 0.44 | 2.50E-04 | 0.39 | 1.37E-03 |
| *FARSA* | 2193 | enzyme | 0.41 | 6.40E-04 | 0.37 | 2.75E-03 |
| *FEN1* | 2237 | enzyme | 0.5 | 4.00E-05 | 0.46 | 1.10E-04 |
| *FERMT2* | 10979 | other | -0.38 | 1.48E-03 | -0.38 | 2.11E-03 |
| *FHL1* | 2273 | other | -0.37 | 2.27E-03 | -0.6 | 6.89E-08 |
| *FHL5* | 9457 | transcription regulator | -0.35 | 3.60E-03 | -0.45 | 1.60E-04 |
| *FIBP* | 9158 | other | 0.36 | 3.22E-03 | 0.42 | 5.80E-04 |
| *FLAD1* | 80308 | enzyme | 0.39 | 1.19E-03 | 0.4 | 1.08E-03 |
| *FNIP1* | 96459 | other | -0.36 | 3.21E-03 | -0.38 | 2.47E-03 |
| *FOXM1* | 2305 | transcription regulator | 0.55 | 1.44E-06 | 0.49 | 3.00E-05 |
| *GAPDH* | 2597 | enzyme | 0.61 | 1.34E-08 | 0.43 | 3.80E-04 |
| *GART* | 2618 | enzyme | 0.44 | 2.30E-04 | 0.35 | 4.87E-03 |
| *GAS7* | 8522 | transcription regulator | -0.35 | 3.74E-03 | -0.4 | 9.70E-04 |
| *GPI* | 2821 | enzyme | 0.37 | 2.39E-03 | 0.41 | 7.90E-04 |
| *GPR116* | 221395 | G-protein coupled receptor | -0.37 | 2.23E-03 | -0.41 | 7.00E-04 |
| *GSTM1* | 2944 | enzyme | -0.38 | 1.66E-03 | -0.37 | 3.15E-03 |
| *GTSE1* | 51512 | other | 0.57 | 5.91E-07 | 0.44 | 2.40E-04 |
| *HCK* | 3055 | kinase | 0.45 | 1.40E-04 | 0.45 | 2.00E-04 |
| *HJURP* | 55355 | other | 0.41 | 7.20E-04 | 0.51 | 1.00E-05 |
| *HN1* | 51155 | other | 0.36 | 2.71E-03 | 0.47 | 6.00E-05 |
| *HSPB6* | 126393 | other | -0.36 | 2.80E-03 | -0.43 | 4.40E-04 |
| *IL1RN* | 3557 | cytokine | 0.38 | 1.88E-03 | 0.36 | 4.30E-03 |
| *IL4I1* | 259307 | enzyme | 0.43 | 3.20E-04 | 0.46 | 1.30E-04 |
| *INCENP* | 3619 | other | 0.36 | 2.70E-03 | 0.36 | 4.05E-03 |
| *IRAK1* | 3654 | kinase | 0.43 | 3.70E-04 | 0.44 | 2.10E-04 |
| *JAM2* | 58494 | other | -0.45 | 1.30E-04 | -0.39 | 1.51E-03 |
| *KCNJ6* | 3763 | ion channel | 0.36 | 3.38E-03 | 0.35 | 5.10E-03 |
| *KIAA1217* | 56243 | other | -0.38 | 1.90E-03 | -0.38 | 1.89E-03 |
| *KIF20A* | 10112 | transporter | 0.46 | 1.20E-04 | 0.35 | 5.15E-03 |
| *KIF2C* | 11004 | other | 0.55 | 1.94E-06 | 0.59 | 1.52E-07 |
| *KIF4A* | 24137 | other | 0.6 | 1.41E-07 | 0.41 | 6.70E-04 |
| *KLF2* | 10365 | transcription regulator | -0.46 | 9.00E-05 | -0.47 | 9.00E-05 |
| *KLHL3* | 26249 | other | -0.47 | 7.00E-05 | -0.41 | 8.10E-04 |
| *LAD1* | 3898 | other | 0.36 | 3.27E-03 | 0.42 | 4.80E-04 |
| *LAMA2* | 3908 | other | -0.39 | 1.12E-03 | -0.49 | 4.00E-05 |
| *LAMB1* | 3912 | other | -0.37 | 2.25E-03 | -0.44 | 2.50E-04 |
| *LDB2* | 9079 | transcription regulator | -0.45 | 1.60E-04 | -0.46 | 1.20E-04 |
| *LDHA* | 3939 | enzyme | 0.37 | 2.15E-03 | 0.35 | 6.20E-03 |
| *LHFP* | 10186 | other | -0.43 | 2.80E-04 | -0.52 | 1.00E-05 |
| *LILRB4* | 11006 | other | 0.35 | 3.54E-03 | 0.49 | 4.00E-05 |
| *LMNB2* | 84823 | other | 0.48 | 5.00E-05 | 0.59 | 9.16E-08 |
| *LONP1* | 9361 | peptidase | 0.39 | 1.08E-03 | 0.35 | 4.87E-03 |
| *LRRC17* | 10234 | other | -0.48 | 4.00E-05 | -0.45 | 1.80E-04 |
| *MAFG* | 4097 | transcription regulator | 0.4 | 9.40E-04 | 0.4 | 9.70E-04 |
| *MAGOH* | 4116 | other | 0.35 | 3.72E-03 | 0.39 | 1.42E-03 |
| *MAMDC2* | 256691 | other | -0.44 | 1.90E-04 | -0.37 | 2.70E-03 |
| *MEG3* | 55384 | other | -0.42 | 5.35E-04 | -0.44 | 2.30E-04 |
| *MFAP4* | 4239 | other | -0.44 | 1.90E-04 | -0.49 | 3.00E-05 |
| *MKI67* | 4288 | other | 0.53 | 4.36E-06 | 0.53 | 3.76E-06 |
| *MRPL4* | 51073 | other | 0.42 | 4.00E-04 | 0.46 | 1.10E-04 |
| *MRPS12* | 6183 | other | 0.42 | 4.40E-04 | 0.41 | 7.60E-04 |
| *MRPS7* | 51081 | other | 0.35 | 3.85E-03 | 0.39 | 1.43E-03 |
| *MSRB3* | 253827 | enzyme | -0.45 | 1.70E-04 | -0.49 | 2.00E-05 |
| *MTHFD1L* | 25902 | enzyme | 0.37 | 2.54E-03 | 0.35 | 5.43E-03 |
| *MTUS1* | 57509 | other | -0.4 | 7.80E-04 | -0.35 | 5.57E-03 |
| *MYADM* | 91663 | other | -0.49 | 2.00E-05 | -0.47 | 9.00E-05 |
| *MYH11* | 4629 | other | -0.61 | 6.67E-08 | -0.4 | 1.32E-03 |
| *NCAPD2* | 9918 | other | 0.47 | 6.00E-05 | 0.51 | 1.00E-05 |
| *NCAPH* | 23397 | other | 0.4 | 8.50E-04 | 0.36 | 3.88E-03 |
| *NEK1* | 4750 | kinase | -0.43 | 3.50E-04 | -0.42 | 5.40E-04 |
| *NOD2* | 64127 | other | 0.35 | 3.89E-03 | 0.39 | 1.65E-03 |
| *NOSTRIN* | 115677 | transcription regulator | -0.43 | 3.60E-04 | -0.38 | 2.03E-03 |
| *NPB* | 256933 | other | 0.36 | 2.73E-03 | 0.38 | 2.37E-03 |
| *OLFML1* | 283298 | other | -0.37 | 2.51E-03 | -0.38 | 2.11E-03 |
| *OSCAR* | 126014 | other | 0.37 | 2.09E-03 | 0.54 | 1.87E-06 |
| *PALLD* | 23022 | other | -0.48 | 6.00E-05 | -0.37 | 2.88E-03 |
| *PDCD5* | 9141 | other | 0.4 | 7.90E-04 | 0.42 | 5.10E-04 |
| *PDCD6IP* | 10015 | other | -0.35 | 3.96E-03 | -0.35 | 5.61E-03 |
| *PDE5A* | 8654 | enzyme | -0.41 | 6.60E-04 | -0.49 | 3.00E-05 |
| *PDGFD* | 80310 | growth factor | -0.4 | 9.70E-04 | -0.51 | 1.00E-05 |
| *PDIA4* | 9601 | enzyme | 0.37 | 2.15E-03 | 0.43 | 3.30E-04 |
| *PER2* | 8864 | transcription regulator | -0.45 | 1.30E-04 | -0.37 | 3.06E-03 |
| *PIGL* | 9487 | enzyme | -0.36 | 2.63E-03 | -0.35 | 5.20E-03 |
| *PJA2* | 9867 | enzyme | -0.43 | 2.70E-04 | -0.35 | 4.89E-03 |
| *PLEKHH2* | 130271 | other | -0.39 | 1.16E-03 | -0.43 | 4.30E-04 |
| *PLSCR4* | 57088 | enzyme | -0.41 | 7.00E-04 | -0.35 | 5.29E-03 |
| *PODN* | 127435 | other | -0.45 | 1.80E-04 | -0.36 | 4.64E-03 |
| *POLA2* | 23649 | enzyme | 0.47 | 7.00E-05 | 0.39 | 1.58E-03 |
| *PPAP2A* | 8611 | phosphatase | -0.37 | 2.18E-03 | -0.39 | 1.39E-03 |
| *PPM1G* | 5496 | phosphatase | 0.38 | 1.80E-03 | 0.38 | 2.54E-03 |
| *PPP1R14B* | 26472 | phosphatase | 0.55 | 1.55E-06 | 0.37 | 2.84E-03 |
| *PRAME* | 23532 | other | 0.38 | 1.81E-03 | 0.38 | 2.18E-03 |
| *PRICKLE2* | 166336 | other | -0.37 | 2.35E-03 | -0.45 | 1.80E-04 |
| *PRKACB* | 5567 | kinase | -0.35 | 3.94E-03 | -0.37 | 3.15E-03 |
| *PSMB9* | 5698 | peptidase | 0.36 | 3.24E-03 | 0.38 | 1.93E-03 |
| *PTPRB* | 5787 | phosphatase | -0.38 | 1.67E-03 | -0.44 | 2.30E-04 |
| *RANBP1* | 5902 | other | 0.41 | 6.80E-04 | 0.47 | 8.00E-05 |
| *RASA1* | 5921 | transporter | -0.37 | 2.38E-03 | -0.37 | 2.76E-03 |
| *RASSF4* | 83937 | other | 0.49 | 3.00E-05 | 0.56 | 5.78E-07 |
| *RBM38* | 55544 | other | 0.54 | 2.05E-06 | 0.39 | 1.72E-03 |
| *RGS5* | 8490 | other | -0.42 | 5.75E-04 | -0.54 | 1.80E-06 |
| *RHOJ* | 57381 | enzyme | -0.47 | 1.95E-04 | -0.53 | 4.09E-06 |
| *RNASE4* | 6038 | enzyme | -0.47 | 8.00E-05 | -0.44 | 2.70E-04 |
| *RNASEH2A* | 10535 | enzyme | 0.36 | 2.94E-03 | 0.4 | 9.90E-04 |
| *RORA* | 6095 | ligand-dependent nuclear receptor | -0.35 | 3.60E-03 | -0.42 | 5.50E-04 |
| *RUNX1T1* | 862 | transcription regulator | -0.39 | 1.07E-03 | -0.39 | 1.65E-03 |
| *SATB2* | 23314 | transcription regulator | -0.4 | 8.50E-04 | -0.39 | 1.44E-03 |
| *SDPR* | 8436 | other | -0.39 | 1.40E-03 | -0.35 | 5.07E-03 |
| *SEMA5A* | 9037 | transmembrane receptor | -0.47 | 7.50E-05 | -0.36 | 4.38E-03 |
| *SFRP2* | 6423 | transmembrane receptor | -0.36 | 3.16E-03 | -0.36 | 4.43E-03 |
| *SGOL1* | 151648 | other | 0.4 | 1.28E-03 | 0.4 | 1.13E-03 |
| *SH3D19* | 152503 | other | -0.39 | 1.32E-03 | -0.41 | 7.20E-04 |
| *SIM2* | 6493 | transcription regulator | 0.38 | 1.45E-03 | 0.37 | 3.23E-03 |
| *SKP2* | 6502 | enzyme | 0.48 | 8.00E-04 | 0.35 | 5.58E-03 |
| *SLC7A5* | 8140 | transporter | 0.39 | 1.27E-03 | 0.43 | 4.00E-04 |
| *SLIT2* | 9353 | other | -0.5 | 2.00E-05 | -0.48 | 4.00E-05 |
| *SMAD5* | 4090 | transcription regulator | -0.42 | 4.10E-04 | -0.41 | 7.10E-04 |
| *SMARCA4* | 6597 | transcription regulator | 0.36 | 3.07E-03 | 0.42 | 4.90E-04 |
| *SMOC2* | 64094 | other | -0.37 | 2.59E-03 | -0.43 | 4.20E-04 |
| *SNRK* | 54861 | kinase | -0.42 | 4.90E-04 | -0.36 | 4.73E-03 |
| *SOD2* | 6648 | enzyme | 0.37 | 2.39E-03 | 0.38 | 2.42E-03 |
| *SPARC* | 6678 | other | -0.39 | 1.30E-03 | -0.36 | 4.41E-03 |
| *SPARCL1* | 8404 | other | -0.44 | 2.40E-04 | -0.46 | 1.20E-04 |
| *SPC25* | 57405 | other | 0.37 | 2.14E-03 | 0.48 | 4.00E-05 |
| *SRGAP1* | 57522 | other | -0.41 | 5.60E-04 | -0.43 | 3.60E-04 |
| *SRPX* | 8406 | other | -0.43 | 2.90E-04 | -0.46 | 9.00E-05 |
| *SSBP2* | 23635 | transcription regulator | -0.35 | 3.74E-03 | -0.44 | 2.70E-04 |
| *SSRP1* | 6749 | transcription regulator | 0.37 | 2.34E-03 | 0.36 | 4.06E-03 |
| *ST14* | 6768 | peptidase | 0.47 | 8.00E-05 | 0.36 | 4.66E-03 |
| *STIP1* | 10963 | other | 0.38 | 1.88E-03 | 0.53 | 1.00E-05 |
| *SUV39H1* | 6839 | enzyme | 0.4 | 8.00E-04 | 0.37 | 3.19E-03 |
| *SVEP1* | 79987 | other | -0.41 | 7.40E-04 | -0.49 | 2.00E-05 |
| *SYNJ2BP* | 55333 | other | -0.35 | 3.58E-03 | -0.4 | 9.50E-04 |
| *SYTL2* | 54843 | other | -0.39 | 1.39E-03 | -0.48 | 5.00E-05 |
| *TEAD4* | 7004 | transcription regulator | 0.46 | 2.95E-04 | 0.43 | 3.70E-04 |
| *TH* | 7054 | enzyme | 0.38 | 1.65E-03 | 0.37 | 3.47E-03 |
| *THBS4* | 7060 | other | -0.51 | 1.00E-05 | -0.46 | 1.20E-04 |
| *TIMP3* | 7078 | other | -0.42 | 4.80E-04 | -0.42 | 5.20E-04 |
| *TKT* | 7086 | enzyme | 0.44 | 2.00E-04 | 0.4 | 1.05E-03 |
| *TMEM119* | 338773 | other | -0.38 | 1.88E-03 | -0.39 | 1.41E-03 |
| *TMEM144* | 55314 | other | -0.36 | 2.71E-03 | -0.37 | 3.47E-03 |
| *TMEM48* | 55706 | other | 0.4 | 8.60E-04 | 0.38 | 1.96E-03 |
| *TOP2A* | 7153 | enzyme | 0.37 | 2.27E-03 | 0.37 | 3.50E-03 |
| *TPI1* | 7167 | enzyme | 0.46 | 4.35E-04 | 0.42 | 5.40E-04 |
| *TPX2* | 22974 | other | 0.52 | 1.00E-05 | 0.37 | 3.50E-03 |
| *TRIP13* | 9319 | transcription regulator | 0.46 | 1.00E-04 | 0.35 | 5.56E-03 |
| *TROAP* | 10024 | peptidase | 0.38 | 1.61E-03 | 0.44 | 2.10E-04 |
| *TSEN54* | 283989 | enzyme | 0.42 | 4.70E-04 | 0.36 | 4.20E-03 |
| *TSHZ2* | 128553 | other | -0.39 | 1.39E-03 | -0.4 | 1.05E-03 |
| *TSHZ3* | 57616 | transcription regulator | -0.42 | 4.60E-04 | -0.47 | 8.00E-05 |
| *TSPAN31* | 6302 | other | -0.42 | 4.50E-04 | -0.43 | 3.70E-04 |
| *TSPAN7* | 7102 | other | -0.44 | 1.38E-03 | -0.37 | 2.63E-03 |
| *UACA* | 55075 | other | -0.39 | 1.38E-03 | -0.35 | 5.14E-03 |
| *UBE2B* | 7320 | enzyme | -0.36 | 3.06E-03 | -0.4 | 1.06E-03 |
| *UBE2C* | 11065 | enzyme | 0.4 | 1.04E-03 | 0.47 | 7.00E-05 |
| *UBL3* | 5412 | other | -0.41 | 6.90E-04 | -0.38 | 1.96E-03 |
| *UQCRH* | 7388 | enzyme | 0.45 | 1.80E-04 | 0.39 | 1.69E-03 |
| *USP39* | 10713 | peptidase | 0.41 | 7.50E-04 | 0.37 | 3.07E-03 |
| *USP5* | 8078 | peptidase | 0.37 | 2.58E-03 | 0.41 | 6.90E-04 |
| *VAC14* | 55697 | other | 0.45 | 1.80E-04 | 0.35 | 4.99E-03 |
| *WDR19* | 57728 | other | -0.42 | 4.50E-04 | -0.36 | 4.04E-03 |
| *WDR67* | 93594 | other | 0.37 | 2.51E-03 | 0.35 | 5.39E-03 |
| *YDJC* | 150223 | other | 0.46 | 1.20E-04 | 0.38 | 2.50E-03 |
| *ZBTB20* | 26137 | transcription regulator | -0.41 | 7.50E-04 | -0.47 | 8.00E-05 |
| *ZNF259* | 8882 | other | 0.45 | 1.40E-04 | 0.35 | 5.31E-03 |
| *ZNF652* | 22834 | other | -0.37 | 2.29E-03 | -0.37 | 2.71E-03 |
| Abbreviation: SUV, standardized uptake value. | | | | | | |

**Table S2.** Prediction of activated upstream regulators in the SHC.

| **Upstream Regulator** | **Exp Log Ratio** | **Molecule Type** | **Predicted Activation State** | **Activation z-score** | ***P*-value*** |
| --- | --- | --- | --- | --- | --- |
| *FOXM1* | 0.916 | transcription regulator | Activated | 4.504 | 6.37 x 10^-20^ |
| *AURKB* | 0.762 | kinase | Activated | 2.236 | 4.08 x 10^-3^ |
| *NCOA3* | 0.145 | transcription regulator | Activated | 2.236 | 4.63 x 10^-3^ |
| *CREB1* | -0.985 | transcription regulator | Activated | 2.236 | 1.07 x 10^-2^ |
| *MITF* |  | transcription regulator | Activated | 4.642 | 3.26 x 10^-13^ |
| *RARA* |  | ligand-dependent nuclear receptor | Activated | 4.146 | 4.88 x 10^-5^ |
| *FOXO1* |  | transcription regulator | Activated | 3.838 | 3.06 x 10^-5^ |
| *ESR1* |  | ligand-dependent nuclear receptor | Activated | 3.795 | 8.93 x 10^-5^ |
| *IL1B* |  | cytokine | Activated | 2.845 | 1.94 x 10^-5^ |
| *E2f* |  | group | Activated | 2.449 | 1.08 x 10^-7^ |
| *Hdac* |  | group | Activated | 2.373 | 5.35 x 10^-2^ |
| *ATF6* |  | transcription regulator | Activated | 2.333 | 4.53 x 10^-6^ |
| *estrogen receptor* |  | group | Activated | 2.236 | 2.94 x 10^-3^ |
| *RUNX2* |  | transcription regulator | Activated | 2.236 | 2.49 x 10^-2^ |
| *S100A6* |  | transporter | Activated | 2.121 | 7.74 x 10^-5^ |
| *MYC* |  | transcription regulator | Activated | 2.062 | 9.40 x 10^-4^ |
| *OGT* |  | enzyme | Activated | 2 | 3.30 x 10^-3^ |
| *ATF3* |  | transcription regulator | Inhibited | -2 | 2.27 x 10^-2^ |
| *CTLA4* |  | transmembrane receptor | Inhibited | -2.121 | 4.17 x 10^-5^ |
| *TGFB1* |  | growth factor | Inhibited | -2.166 | 3.48 x 10^-8^ |
| *E2F6* |  | transcription regulator | Inhibited | -2.236 | 2.60 x 10^-4^ |
| *AR* |  | ligand-dependent nuclear receptor | Inhibited | -2.405 | 8.79 x 10^-7^ |
| *UXT* |  | transcription regulator | Inhibited | -2.429 | 7.67 x 10^-3^ |
| *miR-483-3p (miRNAs w/seed CACUCCU)* |  | mature microRNA | Inhibited | -2.443 | 2.78 x 10^-2^ |
| *CDKN1A* |  | kinase | Inhibited | -3.02 | 6.01 x 10^-3^ |
| *KDM5B* |  | transcription regulator | Inhibited | -3.418 | 3.10 x 10^-6^ |
| *NUPR1* |  | transcription regulator | Inhibited | -3.873 | 5.61 x 10^-9^ |
| *TP53* |  | transcription regulator | Inhibited | -3.952 | 7.56 x 10^-11^ |
| **P*-values were calculated by Fisher’s exact test based on the number of interconnected genes with upstream regulators.  It was predicted that an upstream regulator was activated when the activated *Z*-score was greater than 0, while it was predicted that an upstream regulator was inhibited when the activated *Z*-score was less than 0. This prediction was carried out using Ingenuity Pathway Analysis software.  Abbreviation: SHC, SUV-high-cluster. | | | | | |

**Table S3. Kyoto Encyclopedia of Genes and Genomes (KEGG) pathways associated with metabolism and their involved genes.**

| **Metabolic pathways** | **Involved genes** |
| --- | --- |
| KEGG_FRUCTOSE_AND_MANNOSE_METABOLISM | *MPI, PMM2, PMM1, FBP2, PFKM, GMDS, PFKFB4, PFKL, MTMR6, TPI1, PHPT1, PFKFB3, FUK, PFKFB2, MTMR1, PFKFB1, AKR1B10, FPGT, KHK, FBP1, MTMR2, HK2, HK3, HK1, ALDOA, ALDOC, ALDOB, MTMR7, TSTA3, AKR1B1, SORD, GMPPA, PFKP, GMPPB* |
| KEGG_GALACTOSE_METABOLISM | *GCK, GALK1, GLB1, GALE, B4GALT1, PGM2, LALBA, PFKM, PFKL, MGAM, HK2, HK1, HK3, GALT, G6PC2, GLA, GANC, LCT, GALK2, G6PC, UGP2, PGM1, AKR1B1, B4GALT2, GAA, PFKP* |
| KEGG_ASCORBATE_AND_ALDARATE_METABOLISM | *UGT1A10, UGT1A8, UGT1A7, UGT1A6, ALDH1B1, UGT2B28, ALDH2, UGT1A5, MIOX, UGDH, UGT2A1, ALDH9A1, ALDH3A2, UGT1A9, ALDH7A1, UGT2B11, UGT2B10, UGT2B7, UGT2B4, UGT2A3, UGT1A4, UGT1A1, UGT2B17, UGT1A3, UGT2B15* |
| KEGG_FATTY_ACID_METABOLISM | *CPT1A, CPT1C, ACADS, ALDH1B1, ACADSB, ACADL, ALDH2, ACADM, CYP4A11, ACAT2, ACADVL, ACAT1, ACAA2, HADH, HADHB, HADHA, CYP4A22, ADH7, ADH6, ACSL6, ADH1B, ADH1C, ADH4, ECHS1, ADH5, ALDH9A1, ALDH3A2, ACSL5, ADH1A, EHHADH, GCDH, ALDH7A1, ACOX3, ACSL1, ACAA1, CPT2, CPT1B, ACOX1, ECI2, ECI1, ACSL3, ACSL4* |
| KEGG_PURINE_METABOLISM | *POLR2G, NT5C2, POLR2H, ENPP3, POLR2E, POLR2F, ENPP1, XDH, POLR2I, POLR2J, POLE3, ADSS, PRPS2, ADSL, NME6, POLR1D, PNPT1, POLR2K, PDE11A, POLD3, POLR2L, AK1, NME1-NME2, AK2, AK4, POLA2, CANT1, AMPD1, AMPD3, AMPD2, NME4, GUCY2D, NME1, ATIC, NME2, DGUOK, POLR3C, NME3, POLR3G, POLR3F, PRUNE, C17orf48, PDE4B, PDE4C, PDE3B, PDE4A, NUDT2, GMPR2, PDE6C, PDE6D, PDE4D, PDE6A, PRIM1, PRIM2, AK5, GMPR, NME5, PDE7B, PDE5A, POLD4, GUCY1A2, FHIT, ADCY3, ADCY2, ADCY1, POLA1, PDE3A, PDE2A, PDE1C, ADSSL1, ADA, ENTPD8, POLE2, PDE8A, PDE9A, PDE1B, NUDT9, POLD1, GDA, POLD2, POLE, PDE6B, PDE7A, PRPS1, POLR1E, PAICS, NT5C1A, POLR2B, POLR3A, PDE6H, POLR2A, PDE6G, POLR2D, DCK, POLR2C, ADCY8, ADCY9, ADCY6, AK7, ADCY7, ADCY5, GMPS, ADCY10, POLR3K, POLR1B, PNP, RRM2B, PDE1A, POLR2J2, HPRT1, GART, PAPSS1, PAPSS2, NT5M, ADCY4, IMPDH1, IMPDH2, POLR3H, POLR2J3, POLR1A, POLR3B, GUCY1B3, PKLR, GUCY2C, ENTPD4, PKM2, GUCY1A3, GUK1, GUCY2F, ADK, ITPA, POLR1C, PRHOXNB, POLE4, RRM1, POLR3D, RRM2, NT5C3, PDE8B, POLR3GL, NT5E, NPR2, NPR1, PDE10A, ENTPD6, ENTPD2, ENTPD5, ENTPD3, NUDT5, ENTPD1, PFAS, NT5C1B, APRT, PRPS1L1, PPAT, NME7, NT5C, ALLC, ZNRD1* |
| KEGG_PYRIMIDINE_METABOLISM | *NT5C2, POLR2G, POLR2H, POLR2E, POLR2F, POLR2I, POLR2J, POLE3, TYMS, TXNRD1, NME6, POLR1D, PNPT1, POLR2K, DHODH, POLD3, POLR2L, NME1-NME2, POLA2, UPRT, CANT1, NME4, NME1, NME2, NME3, POLR3C, POLR3G, POLR3F, NUDT2, PRIM1, PRIM2, NME5, POLD4, DTYMK, CTPS, POLA1, ENTPD8, POLE2, POLD1, POLD2, POLE, CAD, CMPK2, POLR1E, NT5C1A, POLR3A, DCTD, POLR2B, POLR2A, DCK, POLR2D, POLR2C, UCK1, UPB1, TXNRD2, CMPK1, POLR3K, RRM2B, PNP, POLR1B, UPP2, CTPS2, POLR2J2, CDA, NT5M, DUT, POLR3H, UCKL1, POLR2J3, POLR1A, POLR3B, ENTPD4, ITPA, POLR1C, POLE4, RRM1, RRM2, POLR3D, NT5C3, DPYS, DPYD, TYMP, UMPS, UCK2, POLR3GL, TK1, NT5E, TK2, UPP1, ENTPD6, ENTPD5, ENTPD3, ENTPD1, NT5C1B, NT5C, NME7, AK3, ZNRD1* |
| KEGG_ALANINE_ASPARTATE_AND_GLUTAMATE_METABOLISM | *GLUD2, GFPT2, AGXT2, CPS1, GLS2, ABAT, ADSS, ADSL, GLUD1, GLS, ASL, IL4I1, GPT, GFPT1, GPT2, DDO, ADSSL1, ACY3, GAD1, GAD2, GOT2, AGXT, GLUL, NIT2, GOT1, ALDH4A1, CAD, ALDH5A1, ASS1, ASNS, PPAT, ASPA* |
| KEGG_GLYCINE_SERINE_AND_THREONINE_METABOLISM | *ALAS1, ALAS2, GLYCTK, MAOB, AGXT2, MAOA, AOC2, GATM, SDS, AOC3, GNMT, SHMT1, GCAT, PHGDH, DMGDH, SHMT2, SRR, PSAT1, BHMT, GLDC, DLD, GAMT, PSPH, AGXT, SARDH, AMT, CHDH, CBS, CTH, PIPOX, DAO* |
| KEGG_CYSTEINE_AND_METHIONINE_METABOLISM | *AMD1, SRM, ADI1, AHCY, DNMT1, SDS, TRDMT1, DNMT3A, DNMT3B, LDHC, IL4I1, SMS, LDHB, ENOPH1, BHMT, GOT2, CDO1, APIP, GOT1, LDHAL6B, TAT, AHCYL1, MTAP, MAT2B, LDHA, MTR, CBS, MAT1A, AHCYL2, CTH, MAT2A, MPST, LDHAL6A, DNMT3L* |
| KEGG_ARGININE_AND_PROLINE_METABOLISM | *SRM, ADC, GLUD2, GLS2, ARG1, ARG2, GLUD1, GLS, ASL, CKMT1A, P4HA2, SMS, CKB, NOS3, NOS2, NOS1, GOT2, GLUL, P4HA1, ABP1, ALDH7A1, LAP3, AGMAT, GOT1, PYCR1, ASS1, ALDH18A1, SAT1, NAGS, DAO, AMD1, MAOB, MAOA, CPS1, P4HA3, ALDH1B1, GATM, ALDH2, OTC, PRODH2, OAT, CKM, ACY1, SAT2, ALDH9A1, ALDH3A2, CKMT1B, PYCRL, GAMT, PRODH, ALDH4A1, CKMT2, PYCR2, ODC1* |
| KEGG_HISTIDINE_METABOLISM | *CNDP1, MAOB, MAOA, ALDH1B1, ALDH2, METTL6, ALDH3A1, WBSCR22, HAL, HNMT, DDC, ACY3, ALDH1A3, METTL2B, ALDH3B1, ALDH3B2, ALDH9A1, ALDH3A2, HEMK1, LCMT2, UROC1, TRMT11, ABP1, ALDH7A1, FTCD, AMDHD1, ASPA, LCMT1, HDC* |
| KEGG_TYROSINE_METABOLISM | *MAOB, MAOA, HPD, AOX1, AOC2, TYR, HGD, METTL6, ALDH3A1, COMT, AOC3, WBSCR22, IL4I1, GSTZ1, DDC, ADH7, DBH, ADH6, METTL2B, ADH1B, ALDH1A3, ADH1C, ALDH3B1, ADH4, ALDH3B2, ADH5, HEMK1, ADH1A, GOT2, LCMT2, TRMT11, GOT1, TYRP1, TAT, MIF, NAT6, PNMT, DCT, LCMT1, TPO, TH, FAH* |
| KEGG_PHENYLALANINE_METABOLISM | *ALDH1A3, ALDH3B1, ALDH3B2, MAOB, MAOA, HPD, GOT2, AOC2, ALDH3A1, AOC3, PAH, GOT1, PRDX6, TAT, MIF, NAT6, IL4I1, DDC* |
| KEGG_TRYPTOPHAN_METABOLISM | *MAOB, MAOA, IDO2, AOX1, ALDH1B1, AANAT, ALDH2, WARS, IDO1, CAT, ACAT2, ACAT1, IL4I1, HADH, OGDH, TPH1, HADHA, DDC, AFMID, CYP1A1, CYP1A2, CYP1B1, ASMT, ECHS1, ALDH9A1, KMO, ALDH3A2, WARS2, EHHADH, OGDHL, GCDH, ALDH7A1, ABP1, INMT, TDO2, HAAO, KYNU, AADAT, TPH2, ACMSD* |
| KEGG_BETA_ALANINE_METABOLISM | *CNDP1, SRM, ECHS1, ALDH9A1, ALDH3A2, GAD1, GAD2, AOC2, ALDH1B1, HIBCH, ABAT, EHHADH, ALDH2, ACADM, UPB1, ALDH7A1, AOC3, DPYS, DPYD, MLYCD, HADHA, SMS* |
| KEGG_TAURINE_AND_HYPOTAURINE_METABOLISM | *GGT7, CSAD, GGT5, GGT1, GAD1, BAAT, GAD2, GGT6, CDO1, ADO* |
| KEGG_SELENOAMINO_ACID_METABOLISM | *METTL6, AHCY, WBSCR22, SEPHS1, GGT1, SEPHS2, METTL2B, HEMK1, GGT6, LCMT2, PAPSS1, PAPSS2, TRMT11, MARS2, SCLY, AHCYL1, GGT7, GGT5, MAT2B, MAT1A, LCMT1, CBS, AHCYL2, CTH, MAT2A, MARS* |
| KEGG_GLUTATHIONE_METABOLISM | *SRM, GGT1, GSTP1, GSTT2, GSTT1, GSTZ1, RRM2B, SMS, PGD, GSTO1, GSTA5, MGST2, LAP3, MGST1, MGST3, GSTA3, GSTM1, GPX7, GSTA4, GPX6, GSTM4, GGCT, GSTM3, GSTM2, GSTM5, GSTA1, GSTA2, GSR, GSS, RRM1, RRM2, GCLC, GSTK1, GPX5, TXNDC12, GPX1, GPX2, GPX3, IDH2, GPX4, IDH1, OPLAH, GCLM, GGT6, ANPEP, GGT7, GSTO2, GGT5, G6PD, ODC1* |
| KEGG_STARCH_AND_SUCROSE_METABOLISM | *ENPP3, GCK, ENPP1, AMY2B, UGT2B28, PGM2, UXS1, UGDH, MGAM, GYS1, GYS2, HK2, HK3, HK1, GBA3, GUSB, TREH, AMY1A, AMY1B, AMY1C, AMY2A, UGT2B11, PYGB, PYGM, PYGL, UGT2A3, UGT1A4, UGT1A1, UGT1A3, UGT1A10, UGT1A8, UGT1A7, UGT1A6, PGM2L1, AGL, UGT1A5, SI, UGT2A1, GBE1, UGT1A9, G6PC2, GANC, G6PC, UGP2, PGM1, UGT2B10, UGT2B7, UGT2B4, GAA, GPI, UGT2B17, UGT2B15* |
| KEGG_AMINO_SUGAR_AND_NUCLEOTIDE_SUGAR_METABOLISM | *MPI, GCK, GFPT2, GALK1, GALE, PMM2, PGM2, PMM1, AMDHD2, GNPDA2, GMDS, UXS1, GNPNAT1, NANS, UGDH, CYB5R3, GFPT1, FUK, NANP, CYB5R1, FPGT, HEXB, HEXA, HK2, HK1, HK3, GALT, GNE, NAGK, CHIT1, UAP1, RENBP, CHIA, NPL, UGP2, GALK2, TSTA3, PGM1, CMAS, PGM3, GNPDA1, GMPPA, GPI, GMPPB* |
| KEGG_GLYCEROLIPID_METABOLISM | *MBOAT2, GPAM, LIPG, DGKZ, DGKE, DGKD, DGKH, MBOAT1, GK, DGAT2, GK2, GLA, ALDH7A1, AGK, DAK, AGPAT3, AWAT2, AGPAT4, AKR1B1, PNLIPRP2, PNLIPRP1, PNLIP, DGAT1, GLYCTK, LPL, ALDH1B1, DGKQ, ALDH2, DGKB, DGKG, AKR1A1, DGKA, PPAP2B, LIPC, DGKI, LCLAT1, AGPAT9, MGLL, AGPAT6, ALDH9A1, ALDH3A2, PNPLA3, PPAP2A, PPAP2C, AGPAT1, AGPAT2, CEL, GPAT2, LIPF* |
| KEGG_INOSITOL_PHOSPHATE_METABOLISM | *PLCB2, PLCB1, INPP1, IPMK, PLCD1, PLCB3, PLCB4, MIOX, TPI1, INPP5A, INPP5B, INPP4A, INPPL1, SYNJ2, PIP4K2B, PLCD3, PIKFYVE, IMPA1, IMPA2, PIP5K1A, PIP5K1B, ISYNA1, INPP5J, INPP5K, SYNJ1, PLCD4, ITPKA, ITPKB, ITPK1, CDIPT, INPP4B, PLCE1, ALDH6A1, PIP5K1C, PIK3C3, PIK3C2B, PIK3C2G, PIP4K2A, PIK3C2A, PTEN, PIP4K2C, MINPP1, IPPK, PIK3CA, PIK3CB, PIK3CD, PLCG1, PLCG2, PLCZ1, PI4KB, PIK3CG, INPP5E, OCRL, PI4KA* |
| KEGG_GLYCEROPHOSPHOLIPID_METABOLISM | *PEMT, PLA2G15, GPD1, GPD1L, LPGAT1, GPAM, MBOAT2, MBOAT7, CHAT, LPCAT1, PTDSS1, ACHE, PLA2G3, DGKZ, ETNK1, DGKE, DGKD, PCYT1A, DGKH, JMJD7-PLA2G4B, CHPT1, MBOAT1, PLA2G6, PLA2G2E, PLA2G10, LPCAT3, PLA2G2A, ETNK2, PLA2G4A, PLA2G5, PLA2G12B, PLA2G4B, PTDSS2, PLA2G2F, PCYT2, CDS1, AGPAT3, AGPAT4, PGS1, LYPLA1, PLA2G1B, C17orf48, CDIPT, DGKQ, CDS2, DGKB, DGKG, PPAP2B, DGKA, DGKI, PHOSPHO1, LCLAT1, TAZ, AGPAT9, GNPAT, LPCAT4, PLA2G4E, AGPAT6, CHKA, PPAP2A, PPAP2C, LYPLA2, PLA2G2C, AGPAT1, PLA2G2D, LPCAT2, PLA2G12A, AGPAT2, PLD1, CHKB, PISD, PLD2, PCYT1B, GPAT2, LCAT, CRLS1, GPD2* |
| KEGG_ETHER_LIPID_METABOLISM | *ENPP2, PAFAH1B1, PPAP2B, LPCAT1, PLA2G3, ENPP6, LPCAT4, PLA2G4E, JMJD7-PLA2G4B, CHPT1, AGPS, PLA2G6, PAFAH1B3, PLA2G2E, PLA2G10, PAFAH2, PLA2G2A, PLA2G4A, PLA2G5, PLA2G12B, PLA2G4B, PLA2G7, PLA2G2F, PPAP2A, PPAP2C, PLA2G2C, PAFAH1B2, PLA2G2D, LPCAT2, PLD1, PLA2G12A, PLD2, PLA2G1B* |
| KEGG_ARACHIDONIC_ACID_METABOLISM | *EPHX2, CYP2E1, ALOX5, CYP4A11, CYP2J2, GGT1, PLA2G3, AKR1C3, PTGIS, ALOX12B, CYP4F2, CYP2C18, PTGES2, ALOX15B, ALOX15, JMJD7-PLA2G4B, PLA2G6, PLA2G2E, PLA2G10, PLA2G2A, PLA2G4A, PLA2G5, PTGS2, PLA2G12B, PTGS1, PLA2G4B, PLA2G2F, ALOX12, CYP2C9, GPX7, GPX6, CYP2C19, CYP2C8, CBR1, CYP2B6, CBR3, CYP2U1, PLA2G1B, LTA4H, PTGES, GPX5, GPX1, CYP4A22, GPX2, GPX3, GPX4, CYP4F3, PLA2G4E, LTC4S, GGT6, GGT7, GGT5, HPGDS, PLA2G2C, PTGDS, PLA2G2D, PLA2G12A, TBXAS1* |
| KEGG_LINOLEIC_ACID_METABOLISM | *CYP2E1, CYP3A5, CYP2J2, CYP3A4, PLA2G3, AKR1B10, CYP2C18, CYP1A2, ALOX15, PLA2G4E, JMJD7-PLA2G4B, PLA2G6, PLA2G2E, PLA2G10, PLA2G2A, PLA2G4A, CYP3A43, PLA2G5, PLA2G12B, PLA2G4B, PLA2G2F, PLA2G2C, PLA2G2D, CYP2C9, PLA2G12A, CYP2C19, CYP2C8, CYP3A7, PLA2G1B* |
| KEGG_ALPHA_LINOLENIC_ACID_METABOLISM | *JMJD7-PLA2G4B, PLA2G6, PLA2G2E, PLA2G10, PLA2G2A, PLA2G4A, PLA2G5, PLA2G12B, PLA2G4B, PLA2G2F, FADS2, ACOX3, PLA2G2C, PLA2G2D, PLA2G3, PLA2G12A, ACOX1, PLA2G4E, PLA2G1B* |
| KEGG_SPHINGOLIPID_METABOLISM | *GAL3ST1, SGPP2, GLB1, GALC, SGMS2, GBA, SPHK2, NEU2, PPAP2B, NEU1, ACER2, UGCG, DEGS2, ARSA, SPHK1, SGPL1, NEU3, SMPD4, SGMS1, ACER3, CERK, SMPD2, ENPP7, DEGS1, SGPP1, NEU4, GLA, ACER1, PPAP2A, ASAH2C, ASAH1, PPAP2C, SMPD1, B4GALT6, SPTLC2, SPTLC1, SMPD3, KDSR, UGT8, ASAH2* |
| KEGG_PYRUVATE_METABOLISM | *ACSS2, PCK2, ME3, GRHPR, PDHB, PCK1, PDHA1, ALDH1B1, PDHA2, HAGH, ALDH2, ACAT2, ACAT1, LDHC, LDHD, HAGHL, ACSS1, LDHB, ACYP2, ME1, ACYP1, ALDH9A1, ALDH3A2, ME2, DLD, MDH2, DLAT, MDH1, ALDH7A1, PC, GLO1, LDHAL6B, LDHA, ACACB, ACACA, PKLR, AKR1B1, LDHAL6A, PKM2, ACOT12* |
| KEGG_GLYOXYLATE_AND_DICARBOXYLATE_METABOLISM | *ACO1, GLYCTK, MTHFD2, GRHPR, CS, HAO1, MDH2, MDH1, MTHFD2L, PGP, MTHFD1L, MTHFD1, HAO2, AFMID, HYI, ACO2* |
| KEGG_PROPANOATE_METABOLISM | *ACSS2, ALDH1B1, ABAT, LOC283398, ALDH2, ACADM, ACAT2, ACAT1, LDHC, MCEE, HADHA, ALDH6A1, ACSS1, LDHB, ECHS1, ALDH9A1, ALDH3A2, PCCB, HIBCH, EHHADH, PCCA, ALDH7A1, LDHAL6B, SUCLG1, MLYCD, LDHA, SUCLA2, ACACB, MUT, SUCLG2, ACACA, ACSS3, LDHAL6A* |
| KEGG_BUTANOATE_METABOLISM | *AACS, PDHB, ALDH1B1, ACADS, PDHA1, ABAT, PDHA2, ALDH2, ACAT2, ACAT1, OXCT1, HADH, BDH1, HADHA, ACSM1, HMGCL, AKR1B10, ECHS1, ALDH9A1, GAD1, ALDH3A2, ACSM3, GAD2, ACSM4, HMGCS2, EHHADH, HMGCS1, ALDH7A1, ALDH5A1, BDH2, OXCT2, ACSM2A, ACSM5, L2HGDH* |
| KEGG_RIBOFLAVIN_METABOLISM | *ACPT, ENPP3, ENPP1, ACP6, RFK, ACPP, FLAD1, TYR, MTMR7, MTMR6, PHPT1, MTMR1, ACP1, ACP2, ACP5, MTMR2* |
| KEGG_NICOTINATE_AND_NICOTINAMIDE_METABOLISM | *NMNAT1, NT5C2, ENPP3, ENPP1, BST1, NNT, AOX1, CD38, NT5M, NADSYN1, NUDT12, NT5C1B, NT5C3, C9orf95, NT5C1A, QPRT, NAMPT, NT5C, NMNAT3, PNP, NMNAT2, NT5E, NADK, NNMT* |
| KEGG_RETINOL_METABOLISM | *RPE65, CYP3A5, UGT2B28, CYP4A11, CYP3A4, RDH8, DHRS3, CYP2C18, ADH1B, ADH1C, ADH4, ADH5, DGAT2, ADH1A, BCMO1, RDH10, CYP26A1, UGT2B11, CYP2C9, AWAT2, CYP26C1, CYP2C19, CYP2C8, CYP2B6, UGT2A3, CYP2A13, UGT1A4, CYP3A7, UGT1A1, UGT1A3, CYP26B1, DHRS9, UGT1A10, DGAT1, UGT1A8, UGT1A7, UGT1A6, ALDH1A1, RETSAT, RDH12, LRAT, RDH11, UGT1A5, CYP2A6, CYP2A7, CYP4A22, DHRS4, CYP1A1, CYP1A2, ADH7, ADH6, UGT2A1, ALDH1A2, CYP3A43, DHRS4L2, RDH16, UGT1A9, RDH5, UGT2B10, UGT2B7, PNPLA4, UGT2B4, UGT2B17, UGT2B15* |
| KEGG_PORPHYRIN_AND_CHLOROPHYLL_METABOLISM | *ALAS1, COX10, UGT1A10, ALAS2, UGT1A8, UGT1A7, ALAD, UGT1A6, CPOX, BLVRA, UGT2B28, BLVRB, UGT1A5, UROS, HCCS, UGT2A1, GUSB, FTMT, UGT1A9, MMAB, FECH, HMOX1, HMOX2, UGT2B11, PPOX, FTH1, UGT2B10, UGT2B7, UROD, UGT2B4, COX15, HMBS, CP, UGT2A3, UGT1A4, EARS2, UGT2B17, UGT1A1, EPRS, UGT2B15, UGT1A3* |
| KEGG_NITROGEN_METABOLISM | *CA5A, CA5B, CA6, CA12, CA7, GLUD2, CA2, CPS1, CA3, CA4, GLS2, GLUL, CA8, CA14, GLUD1, CA9, CA1, GLS, AMT, HAL, ASNS, CA13, CTH* |
| KEGG_SULFUR_METABOLISM | *SULT1E1, PAPSS1, SULT1A4, SULT1A3, PAPSS2, CHST11, SULT1A1, SULT2B1, SUOX, BPNT1, CHST13, SULT1A2, CHST12* |
| KEGG_METABOLISM_OF_XENOBIOTICS_BY_CYTOCHROME_P450 | *CYP2F1, CYP2E1, AKR1C4, EPHX1, CYP3A5, UGT2B28, CYP3A4, GSTP1, GSTT2, GSTT1, AKR1C3, GSTZ1, CYP2C18, ADH1B, CYP2S1, ADH1C, ADH4, ADH5, GSTO1, ADH1A, GSTA5, MGST2, MGST1, MGST3, GSTA3, GSTM1, UGT2B11, CYP2C9, GSTA4, CYP2C19, GSTM4, CYP2C8, GSTM3, CYP2B6, GSTM2, UGT2A3, UGT1A4, UGT1A1, CYP3A7, UGT1A3, GSTM5, UGT1A10, UGT1A8, UGT1A7, GSTA1, UGT1A6, GSTA2, ALDH3A1, UGT1A5, GSTK1, AKR1C2, AKR1C1, CYP1A1, ADH7, CYP1A2, CYP1B1, ADH6, UGT2A1, ALDH1A3, ALDH3B1, ALDH3B2, CYP3A43, UGT1A9, DHDH, GSTO2, UGT2B10, UGT2B7, UGT2B4, UGT2B17, UGT2B15* |
| KEGG_DRUG_METABOLISM_CYTOCHROME_P450 | *CYP2E1, CYP3A5, UGT2B28, CYP3A4, GSTP1, GSTT2, GSTT1, GSTZ1, CYP2C18, CYP2D6, ADH1B, FMO4, ADH1C, ADH4, ADH5, GSTO1, ADH1A, GSTA5, MGST2, MGST1, FMO1, FMO2, MGST3, FMO3, GSTA3, GSTM1, UGT2B11, CYP2C9, FMO5, GSTA4, CYP2C19, GSTM4, CYP2C8, GSTM3, CYP2B6, GSTM2, UGT2A3, CYP2A13, UGT1A4, UGT1A1, CYP3A7, UGT1A3, GSTM5, UGT1A10, UGT1A8, UGT1A7, GSTA1, UGT1A6, MAOB, GSTA2, MAOA, AOX1, ALDH3A1, UGT1A5, GSTK1, CYP2A6, CYP2A7, ADH7, CYP1A2, ADH6, UGT2A1, ALDH1A3, ALDH3B1, ALDH3B2, CYP3A43, UGT1A9, GSTO2, UGT2B10, UGT2B7, UGT2B4, UGT2B17, UGT2B15* |
| KEGG_DRUG_METABOLISM_OTHER_ENZYMES | *XDH, CYP3A5, UGT2B28, UPB1, CYP3A4, GMPS, UPP2, HPRT1, CDA, GUSB, IMPDH1, IMPDH2, CES2, UCKL1, UGT2B11, TPMT, NAT1, CES5A, CYP2A13, UGT2A3, UGT1A4, CYP3A7, UGT1A1, UGT1A3, UGT1A10, UGT1A8, UGT1A7, ITPA, UGT1A6, UGT1A5, CYP2A6, CYP2A7, DPYS, TYMP, DPYD, UMPS, UCK2, TK1, TK2, UPP1, UGT2A1, CYP3A43, CES1, UGT1A9, NAT2, UGT2B10, UGT2B7, UGT2B4, UCK1, UGT2B17, UGT2B15* |
| KEGG_CITRATE_CYCLE_TCA_CYCLE | *IDH3B, DLST, PCK2, CS, PDHB, PCK1, PDHA1, LOC642502, PDHA2, LOC283398, FH, SDHD, OGDH, SDHB, IDH3A, SDHC, IDH2, IDH1, ACO1, ACLY, MDH2, DLD, MDH1, DLAT, OGDHL, PC, SDHA, SUCLG1, SUCLA2, SUCLG2, IDH3G, ACO2* |
| KEGG_PENTOSE_PHOSPHATE_PATHWAY | *RPE, RPIA, PGM2, PGLS, PRPS2, FBP2, PFKM, PFKL, TALDO1, TKT, FBP1, TKTL2, PGD, RBKS, ALDOA, ALDOC, ALDOB, H6PD, LOC729020, PRPS1L1, PRPS1, DERA, G6PD, PGM1, TKTL1, PFKP, GPI* |
| KEGG_PENTOSE_AND_GLUCURONATE_INTERCONVERSIONS | *UGT1A10, UGT1A8, RPE, UGT1A7, UGT1A6, UGT2B28, UGT1A5, CRYL1, UGDH, UGT2A1, GUSB, UGT1A9, DCXR, LOC729020, DHDH, UGT2B11, UGP2, XYLB, UGT2B10, AKR1B1, UGT2B7, UGT2B4, UGT2A3, UGT1A4, UGT2B17, UGT1A1, UGT1A3, UGT2B15* |
| KEGG_OXIDATIVE_PHOSPHORYLATION | *ATP6V1G1, UQCR10, NDUFA5, NDUFA4, COX6CP3, PPA2, ATP5J2, NDUFS7, CYTB, ATP6V0A1, ATP6V1G2, ATP6V0B, ATP5O, COX2, COX1, ATP6AP1, COX8C, COX3, COX5B, COX4I1, ATP6V0A4, ATP12A, ATP6, ATP8, ATP5H, NDUFA2, NDUFA3, NDUFA1, LOC100133737, ATP5G1P5, UQCRQ, ATP6V1H, ATP5D, LHPP, ATP6V0D2, COX7A2L, ATP5G3, ATP6V1C2, ATP5G2, ATP5G1, ATP5F1, ATP5E, COX5A, TCIRG1, UQCRHL, ATP4B, ATP6V1D, ATP4A, ATP5A1, ATP5J, ATP5I, SDHA, ATP6V1A, ATP6V1E1, NDUFA4L2, ATP6V1B2, ATP6V1B1, ATP6V1C1, ATP6V0C, COX10, COX8A, COX7C, LOC642502, SDHD, SDHB, SDHC, ATP6V1G3, COX7B, COX7A1, COX7A2, COX6C, COX17, ATP5B, COX6B1, ATP5C1, LOC644310, ATP6V1F, COX7B2, ATP6V0A2, UQCRB, ATP6V0D1, COX6A2, COX6A1, UQCRH, UQCRFS1, UQCRC2, UQCRC1, NDUFB3, NDUFB1, NDUFB2, NDUFA10, NDUFAB1, ATP6V0E2, ND6, NDUFA9, ND5, NDUFA7, NDUFA8, UQCR11, NDUFA6, NDUFV3, ATP6V1E2, ND4, ND4L, LOC727947, ND2, COX6B2, PPA1, ND3, COX4I2, ND1, ATP5L, ATP6V0E1, NDUFA11, NDUFB10, NDUFS8, NDUFC1, NDUFC2, NDUFS1, NDUFV2, NDUFB6, NDUFS4, NDUFB7, NDUFV1, NDUFB8, NDUFS6, NDUFB9, NDUFS5, NDUFS2, NDUFB4, NDUFS3, NDUFB5, CYC1, COX11, COX15* |
| KEGG_GLYCOLYSIS_GLUCONEOGENESIS | *ACSS2, GCK, PGK2, PGK1, PDHB, PDHA1, PDHA2, PGM2, TPI1, ACSS1, FBP1, ADH1B, HK2, ADH1C, HK1, HK3, ADH4, PGAM2, ADH5, PGAM1, ADH1A, ALDOC, ALDH7A1, LDHAL6B, PKLR, LDHAL6A, ENO1, PKM2, PFKP, BPGM, PCK2, PCK1, ALDH1B1, ALDH2, ALDH3A1, AKR1A1, FBP2, PFKM, PFKL, LDHC, GAPDH, ENO3, ENO2, PGAM4, ADH7, ADH6, LDHB, ALDH1A3, ALDH3B1, ALDH3B2, ALDH9A1, ALDH3A2, GALM, ALDOA, DLD, DLAT, ALDOB, G6PC2, LDHA, G6PC, PGM1, GPI* |
| KEGG_GLYCOSAMINOGLYCAN_DEGRADATION | *HS3ST3A1, HPSE, HPSE2, GLB1, GUSB, HYAL3, GNS, HYAL4, HYAL1, HYAL2, SPAM1, HGSNAT, GALNS, IDUA, HS3ST3B1, SGSH, HEXB, NAGLU, HEXA, IDS, ARSB* |
| KEGG_GLYCOSAMINOGLYCAN_BIOSYNTHESIS_CHONDROITIN_SULFATE | *DSE, B3GALT6, CHPF, XYLT1, CHSY3, XYLT2, CHST14, B3GAT1, CHSY1, CHST11, CHPF2, CHST15, B4GALT7, CHST7, UST, CHST13, CSGALNACT2, B3GAT2, CSGALNACT1, CHST3, CHST12, B3GAT3* |
| KEGG_GLYCOSAMINOGLYCAN_BIOSYNTHESIS_KERATAN_SULFATE | *CHST2, B3GNT1, B3GNT2, CHST1, B4GALT1, B3GNT7, ST3GAL2, B4GALT3, ST3GAL1, B4GALT2, B4GALT4, CHST4, ST3GAL3, FUT8, CHST6* |
| KEGG_GLYCOSAMINOGLYCAN_BIOSYNTHESIS_HEPARAN_SULFATE | *HS3ST5, EXT2, EXT1, NDST3, XYLT1, XYLT2, B3GAT1, NDST1, GLCE, HS3ST3B1, HS2ST1, HS6ST2, B3GAT3, HS3ST1, B3GALT6, HS3ST2, HS6ST1, HS3ST3A1, NDST2, NDST4, HS6ST3, B4GALT7, B3GAT2, EXTL1, EXTL2, EXTL3* |
| KEGG_GLYCOSYLPHOSPHATIDYLINOSITOL_GPI_ANCHOR_BIOSYNTHESIS | *PIGP, PIGW, PIGM, PIGN, PIGS, PIGF, DPM2, PIGQ, PIGH, PIGO, PIGG, PIGZ, GPAA1, PIGU, PGAP1, PIGT, PIGK, PIGY, PIGL, PIGV, PIGB, PIGX, PIGA, GPLD1, PIGC* |
| KEGG_GLYCOSPHINGOLIPID_BIOSYNTHESIS_LACTO_AND_NEOLACTO_SERIES | *B3GNT4, B3GNT2, ST3GAL6, B4GALT1, B3GNT5, FUT9, FUT7, FUT6, FUT5, FUT4, FUT3, FUT2, FUT1, B3GNT1, GCNT2, B3GNT3, B3GALT5, ABO, B4GALT3, B4GALT2, ST3GAL4, B4GALT4, ST3GAL3, B3GALT2, B3GALT1, ST8SIA1* |
| KEGG_GLYCOSPHINGOLIPID_BIOSYNTHESIS_GLOBO_SERIES | *A4GALT, B3GALT5, GLA, FUT9, ST3GAL2, GBGT1, ST3GAL1, FUT2, FUT1, HEXB, NAGA, ST8SIA1, B3GALNT1, HEXA* |
| KEGG_GLYCOSPHINGOLIPID_BIOSYNTHESIS_GANGLIO_SERIES | *ST6GALNAC4, ST6GALNAC3, ST6GALNAC6, B4GALNT1, GLB1, ST6GALNAC5, SLC33A1, ST3GAL5, ST3GAL2, ST3GAL1, HEXB, ST8SIA1, B3GALT4, ST8SIA5, HEXA* |

**Table S4. Primer sequences of the genes for qRT-PCR analysis.**

| **Primer name** | **Primer Sequence** | **Primer length** | **Product size** |
| --- | --- | --- | --- |
| *Actin* F | 5’ CCC TGG AGA AGA GCT ACG AG 3’ | 20 mer | 132 bp |
| *Actin* R | 5’ AGG TAG TTT CGT GGA TGC CA 3’ | 20 mer |  |
| *FOXM1* F | 5’ TGC AGC TAG GGA TGT GAA TCT TC 3’ | 23 mer | 157 bp |
| *FOXM1* R | 5’ GGA GCC CAG TCC ATC AGA ACT 3’ | 21 mer |  |
| *LDHA* F | 5’ GGG GAA CAT GGA GAT TCC AG 3’ | 20 mer | 135 bp |
| *LDHA* R | 5’ AAC CAC CTG CTT GTG AAC C 3’ | 19 mer |  |
| Abbreviations: F, forward; R, reverse. | | | |
